# The Hsp90 machinery facilitates the transport of diphtheria toxin into human cells

**DOI:** 10.1038/s41598-017-00780-x

**Published:** 2017-04-04

**Authors:** Manuel Schuster, Leonie Schnell, Peter Feigl, Carina Birkhofer, Katharina Mohr, Maurice Roeder, Stefan Carle, Simon Langer, Franziska Tippel, Johannes Buchner, Gunter Fischer, Felix Hausch, Manfred Frick, Carsten Schwan, Klaus Aktories, Cordelia Schiene-Fischer, Holger Barth

**Affiliations:** 1 Institute of Pharmacology and Toxicology, University of Ulm Medical Center, Ulm, Germany; 2 Munich Center for Integrated Protein Science and Department Chemistry, Technical University of Munich, Munich, Germany; 3 Max Planck Research Unit for Enzymology of Protein Folding Halle, Halle, Saale Germany; 4 Institute for Organic Chemistry and Biochemistry, Technical University Darmstadt, Darmstadt, Germany; 5 Department of Translational Research in Psychiatry, Max Planck Institute of Psychiatry, Munich, Germany; 6 Institute of General Physiology, University of Ulm, Ulm, Germany; 7 Institute of Experimental and Clinical Pharmacology and Toxicology, University of Freiburg, 79104 Freiburg, Germany; 8 Institute for Biochemistry and Biotechnology, Martin Luther University Halle-Wittenberg, Halle, Saale Germany

## Abstract

Diphtheria toxin kills human cells because it delivers its enzyme domain DTA into their cytosol where it inhibits protein synthesis. After receptor-mediated uptake of the toxin, DTA translocates from acidic endosomes into the cytosol, which might be assisted by host cell factors. Here we investigated the role of Hsp90 and its co-chaperones during the uptake of native diphtheria toxin into human cells and identified the components of the Hsp90 machinery including Hsp90, Hsp70, Cyp40 and the FK506 binding proteins FKBP51 and FKBP52 as DTA binding partners. Moreover, pharmacological inhibition of the chaperone activity of Hsp90 and Hsp70 and of the peptidyl-prolyl *cis/trans* isomerase (PPIase) activity of Cyps and FKBPs protected cells from intoxication with diphtheria toxin and inhibited the pH-dependent trans-membrane transport of DTA into the cytosol. In conclusion, these host cell factors facilitate toxin uptake into human cells, which might lead to development of novel therapeutic strategies against diphtheria.

## Introduction

Diphtheria toxin (DT) produced by *Corynebacterium diphtheriae* is one of the best investigated bacterial AB-type protein toxins (for review see ref. 1) and the causative pathogenicity factor for diphtheria, a severe disease with increasing prevalence in various countries and high lethality, in particular for children. Treatment of diphtheria is achieved by administration of antibiotics targeting the bacteria and with antibodies that neutralize DT in the blood stream before it enters cells. The single-chain DT (58 kDa) consists of the enzymatically active domain DTA (21 kDa), an ADP-ribosyltransferase, in the N-terminal part^2^ and the transport domain DTB (37 kDa) in the C-terminal part. DTB mediates the transport of DTA into the cytosol of target cells, where DTA catalyzes the covalent transfer of ADP-ribose from NAD^+^ onto diphthamide, a modified histidine residue, of elongation factor 2 (EF-2)^3, 4^. Eventually, the mono-ADP-ribosylation of EF-2 leads to the inhibition of protein synthesis and apoptosis. DT-treated HeLa cells round up, which is a specific and highly sensitive endpoint to monitor DTA uptake into the cytosol^5, 6^.

During DT uptake into target cells, the DTB first mediates the binding of DT to cells via its receptor binding (R-) domain and subsequently fosters transport of DTA from acidified endosomes into the cytosol^7^ via its translocation (T-) domain^8^. The R-domain binds to the heparin-binding epidermal growth factor-like growth factor (HB-EGF)^9–11^ and triggers receptor-mediated endocytosis of cell-bound DT. DT is cleaved (nicked) by the cell-associated furin protease on the cell surface as well as in endosomal vesicles^12^ but the DTA and DTB remain linked via an inter-chain disulfide bond between Cys-186 in DTA and Cys-201 in the DTB^13^, which is essential for DTB-mediated transport of DTA into the host cell cytosol^12, 14, 15^. DTA is transported from the luminal side of endosomal membranes to the cytosolic side and this translocation step from early acidified endosomes into the cytosol is the last step during the cellular uptake of the toxin^16–18^. This step is fostered by the T-domain, which forms a pore in the endosomal membrane^19–21^ and thereby mediates trans-membrane transport of unfolded DTA^22–26^. During or after the trans-membrane transport the DTA is released from the DTB by reduction of the disulfide bond by the thioredoxin reductase/thioredoxin system of host cells^6, 16, 27, 28^.

Thioredoxin reductase and heat shock protein (Hsp) 90 were found to be components of a cytosolic translocation factor (CTF) complex, which binds the DTA at the cytosolic side of purified early endosomes and facilitates the trans-membrane transport of a DTA-containing fusion toxin from pre-loaded endosomal vesicles to the cytosol *in vitro* ^29^. We demonstrated that Hsp90 directly binds the DTA *in vitro* and that pharmacological inhibition of its chaperone activity by radicicol (Rad) prevents translocation of a recombinant DTA-containing fusion toxin (LF_N_DTA) from early endosomes into the cytosol *in vitro* and in living cells, thereby protecting cells from intoxication^30^. The LF_N_DTA exploits the transport component of the anthrax toxin (protective antigen, PA63) for artificial delivery across the membranes of acidic endosomes^31^. Cyclosporine A (CsA), a specific pharmacological inhibitor of cyclophilins (Cyps)^32^, showed comparable effects as Rad, suggesting that in addition to Hsp90, Cyps are involved in the uptake of DTA into the host cell cytosol^30^. Moreover, we identified CypA as a novel binding partner of DTA and demonstrated that CypA also facilitates the PA63-mediated translocation of LF_N_DTA across endosomal membranes^30^. Because the used inhibitor CsA is not CypA-selective, further Cyps might be involved in DT uptake. Cyps are members of the peptidyl-prolyl *cis/trans* isomerase (PPIase) family, able to catalyze rate-limiting isomerization reactions in protein folding^33–36^. Interestingly, some PPIases such as Cyp40 and the FK506 binding proteins (FKBP) 51 and 52 are co-chaperones of Hsp90 and components of cellular Hsp90 chaperone complexes^37, 38^.

As all earlier studies addressing the role of host cell chaperones for DTA transport across cell membranes were performed with recombinant fusion toxins, it is still unknown whether Hsp90 and other host cell chaperones and/or PPIases play a role in the uptake of native DT into cells, as suggested earlier^18^. Precisely this important open question was addressed in the present study. By performing different biochemical and biophysical approaches, we identified Hsp70/Hsc70, Cyp40, FKBP51 and FKBP52 as specific binding partners of DTA, in addition to Hsp90 and CypA. Moreover, we exploited novel tailored inhibitors of Hsp70 and the individual PPIases to confirm that the targeted pharmacological inhibition of Hsp70, intracellular Cyps and FKBP51 protected cultured human cells from intoxication with native DT and inhibited the trans-membrane transport of the DTA into the cytosol.

## Results

### In addition to Hsp90 and CypA, DTA binds to Hsp70, Hsc70, Cyp40, FKBP51 and FKBP52

**Figure 1:**
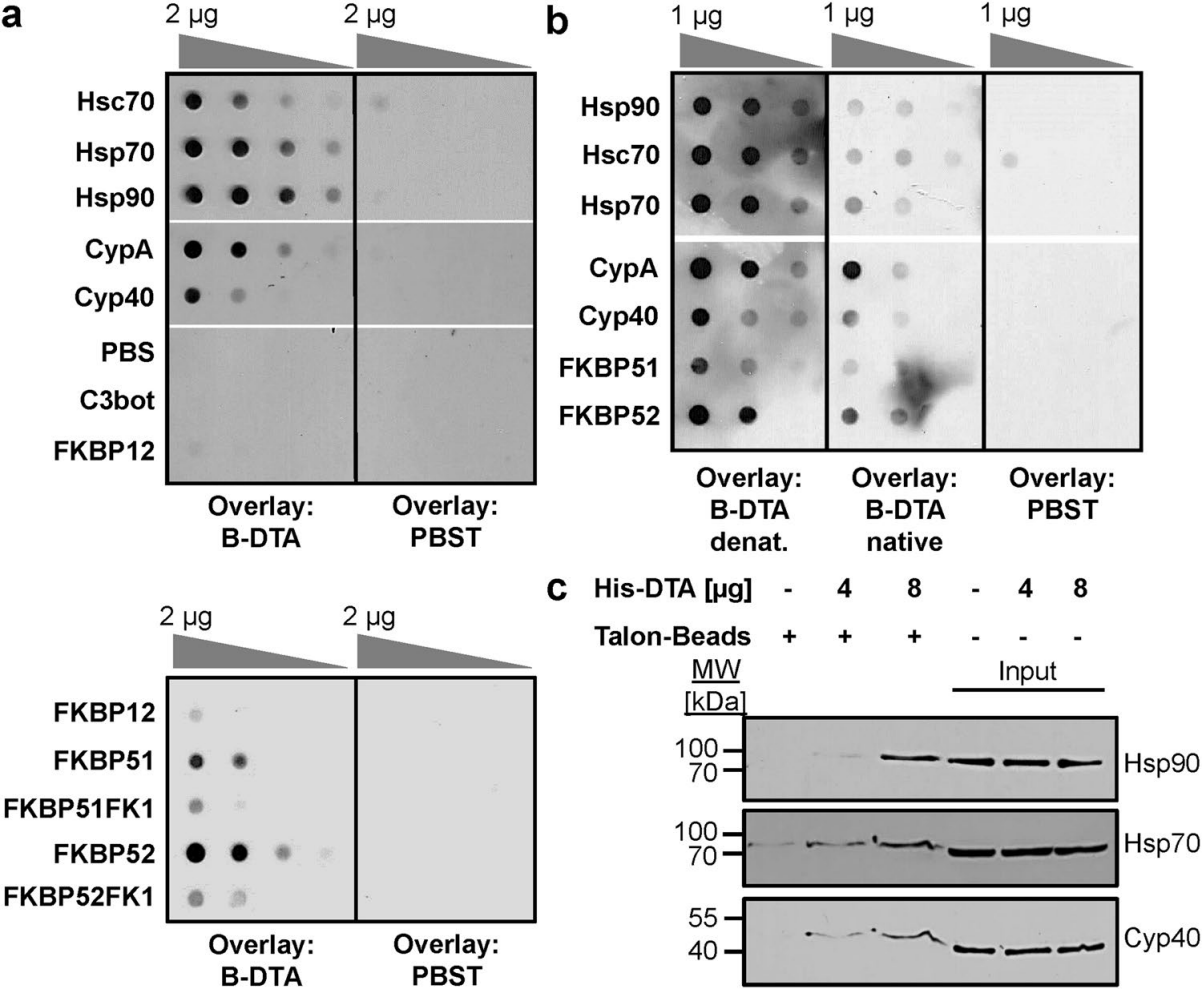
*In vitro*, the enzymatic domain of DT (DTA) specifically binds to the purified recombinant proteins Hsp90, Hsp70, Hsc70, CypA, Cyp40, FKBP51, and FKBP52 and the isolated PPIase domains of FKBP51 and FKBP52. (**a**) Dot blot analysis of the interaction between DTA and recombinant chaperones and PPIases. A serial dilution (2 µg, 1 µg, 500 ng and 250 ng) of each of the indicated recombinant proteins was immobilized on a nitrocellulose membrane by vacuum aspiration with the dot blot system and the transfer confirmed by protein staining with Ponceau S (Supplementary Fig. 1). After blocking of the membrane with 5% non-fat dry milk in PBST, the membrane was cut into two identical portions. With one portion, an overlay with biotinylated DTA in PBST (B-DTA, 9.5 nM) was performed for 1 h. After washing, the bound biotin-DTA was detected with streptavidin-peroxidase and the ECL system. For control, the other portion was incubated with PBST instead of B-DTA and then treated exactly as the first portion. The direct binding of DTA to the PPIase domains of FKBP51 (FKBP51FK1) and FKBP52 (FKBP52FK1) was tested exactly as described before. (**b**) Dot blot analysis of the binding of denatured and native DTA to immobilized chaperones and PPIases. The comparable experiment as described in A was performed with denatured B-DTA in direct comparison to native B-DTA in the overlay. Therefore, DTA was incubated for 1 h at RT either with PBS (native B-DTA) or with 6 M guanidine hydrochloride to obtain denatured B-DTA. (**c**) Co-precipitation of His_6_-DTA with Hsp90, Hsp70 and Cyp40 from cell lysate. HeLa lysate (1,500 µg) was incubated for 30 min at RT with His_6_-DTA (4 µg and 8 µg) or without DTA for control. The subsequent pull-down of His_6_-DTA was performed by incubation with Talon^®^ CellThru beads for 1 h at 4 °C. After extensive washing of beads, the samples were boiled with SDS-sample buffer, followed by Western blot analysis of co-precipitated proteins with specific antibodies against Hsp90, Hsp70 and Cyp40. The blot membranes were cut prior to antibody incubation to detect the respective proteins. Equal protein loading was confirmed by input control which was taken from the respective sample prior to bead incubation.

Prompted by our observation that recombinant DTA directly binds to immobilized Hsp90 and CypA *in vitro* ^30^, dot blotting was performed to test whether the DTA interacts with additional host cell chaperones and PPIases. In addition to Hsp90 and CypA, the DTA bound to immobilized Hsp70, Hsc70, Cyp40, FKBP51 and FKBP52, as shown in the dot blot analysis in Fig. 1a. The binding of DTA to these host cell factors was specific since DTA did not bind to FKBP12 or the non-relevant C3bot protein from *Clostridium botulinum* under equal conditions. Purified DTA also bound to the isolated PPIase domains of FKBP51 and FKBP52, FKBP51FK1 and FKBP52FK1, respectively (Fig. 1a). However, this interaction was weaker compared to the binding of DTA to full-length FKBP51 and FKBP52, suggesting that the rest of these FKBPs might stabilize the binding of DTA to the PPIase domain. The binding of DTA to the recombinant chaperones and PPIases was stronger when DTA was treated with guanidine hydrochloride (Fig. 1b), implicating that (at least partially) unfolded DTA shows a stronger interaction with these host cell factors. It is noteworthy that after longer exposition times the binding of native DTA to all of the spotted chaperones and PPIases became also detectable, comparable to the result shown in the upper panel of Fig. 1a. In all dot blot experiments comparable amounts of immobilized proteins were analyzed, as confirmed by Ponceau S staining (Supplementary Fig. 1).

The direct and specific binding of the DTA to the various purified chaperones and PPIases was confirmed and quantified by isothermal titration calorimetry (ITC, Supplementary Fig. 2, Supplementary Table 1). Binding of each of the PPIases CypA, Cyp40, FKBP51 and FKBP52 to DTA was mainly driven entropically, whereas enthalpy contributed relatively strongly to the formation of the Hsp70/DTA complex.

Furthermore, Hsp90, Hsp70 and Cyp40 co-precipitated with DTA from cell lysate (Fig. 1c). To this end, HeLa lysate was incubated with His_6_-tagged DTA and co-precipitated proteins were analyzed in Western blot analysis with specific antibodies against Hsp90, Hsp70 and Cyp40 (Fig. 1c). Concentration-dependent binding of each of these proteins to DTA was observed while only negligible signals were detectable in co-precipitates from untreated cell lysate.

Taken together, Hsp70, Hsc70, Cyp40, FKBP51 and FKBP52 were identified as novel specific binding partners of DTA *in vitro* in addition to the previously identified host cell factors Hsp90 and CypA. In the following, it was investigated whether these factors are actively involved in the uptake of DT into human cells.

### Pharmacological inhibition of Hsp90, Cyps and FKBPs protects cells from intoxication with DT

**Figure 2:**
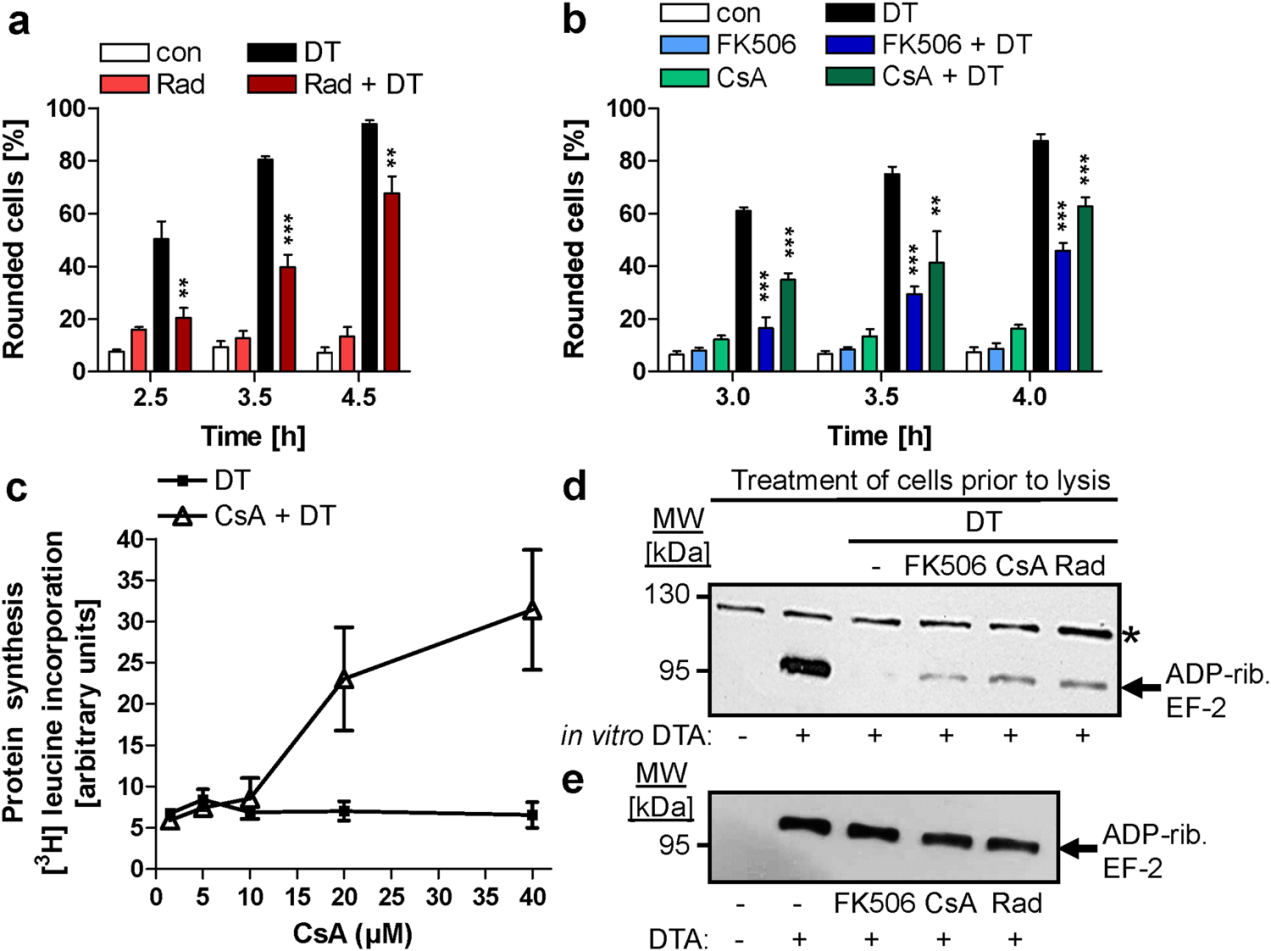
Pharmacological inhibition of Hsp90, Cyps or FKBPs delays intoxication of cells with DT. (**a**) Effect of Rad on intoxication of with DT. HeLa cells were pre-incubated (30 min, 37 °C) with Rad (20 µM) to inhibit Hsp90 or left untreated for control (con). DT (3.4 nM) was added and the DT-induced cell-rounding monitored over time. For control, cells were incubated with Rad alone. The percentages of rounded cells were determined from pictures (Supplementary Fig. 4a) and given as mean ± SD (n = 3). Significance was tested between cells treated with DT in the absence (black bars) and presence of Rad (dark red bars) using Student’s t-test (**p < 0.01, ***p < 0.001). (**b**) The comparable experiment was performed with CsA (10 µM) and FK506 (10 µM). Representative pictures in Supplementary Fig. 4b. (**c**) Effect of CsA on the DT-mediated inhibition of protein biosynthesis in CHO-K1 cells. Cells were incubated for 30 min at 37 °C with the indicated concentrations of CsA or without CsA for control. DT (3.4 nM) was added, cells were further incubated, and after 2 h, the medium was replaced by L-leucine-free medium supplemented with [^3^H]-leucine. After an additional h cells were lysed and proteins precipitated by 10% trichloroacetic acid for measuring the amount of incorporated [^3^H]-leucine by scintillation counting (mean ± SD; n = 2). (**d**) ADP-ribosylation status of EF-2 in HeLa cells treated with DT in the presence or absence of Rad, CsA and FK506. HeLa cells were pre-incubated for 30 min with Rad (20 µM), CsA (20 µM) or FK506 (10 µM) or left untreated. DT (3.4 nM) was added and cells were incubated for 2 h. For control, cells were incubated without DT. Cells were lysed and equal protein amounts incubated for 30 min at 37 °C with biotin-NAD^+^ (10 µM) and DTA (300 ng). Biotinylated (i.e. ADP-ribosylated) proteins were detected by Western blotting with streptavidin-peroxidase to analyze the ADP-ribosylation of EF-2. The asterisk indicates a cell protein that confirms comparable protein loading. The Western blot panel was cropped for presentation purposes only. (**e**) Effect of Rad, CsA and FK506 on DTA activity *in vitro*. HeLa protein (10 µg) was treated (30 min, 37 °C) with either Rad (20 µM), CsA (20 µM) or FK506 (10 µM) or left untreated. DTA (100 ng) and biotin-NAD^+^ (10 µM) were added and ADP-ribosylated EF-2 detected as before. The Western blot panel was cropped for presentation purposes only.

To test whether the identified DTA-binding chaperones and PPIases are involved in the cellular uptake of native DT, HeLa cells were pre-treated with specific pharmacological inhibitors against these factors and afterwards challenged with DT. DT-induced cell-rounding in the absence or presence of the inhibitors was analyzed as highly sensitive and specific endpoint to monitor the cell intoxication process as it clearly indicates the presence of enzymatically active DTA in the host cell cytosol. We confirmed that the DT-induced cell-rounding depends on the presence of enzymatically active DTA in the cytosol by several approaches: i) the enzymatic inactive DT-mutant CRM197 did not cause cell-rounding (not shown); ii) the DT-induced cell-rounding correlated with the ADP-ribosylation status of EF-2 in the cytosol of DT-treated cells (see also Fig. 2d); iii) treatment of cells with bafilomycin (Baf) A1, which prevents endosomal acidification, prevented the rounding of DT-treated cells (see also Supplementary Fig. 6a) and the uptake of DTA into their cytosol.

To investigate whether Hsp90 plays a role for the uptake of DT, HeLa cells were pre-incubated for 30 min with the Hsp90 inhibitor Rad and subsequently challenged with DT. For control, cells were left untreated or incubated with DT in the absence of Rad. The DT-induced cell-rounding was clearly delayed in the presence of Rad (Fig. 2a and Supplementary Fig. 3a), suggesting that Hsp90 is involved in the cytotoxic mode of action of DT. Treatment of cells with Rad in the absence of DT displayed no relevant effect on cell morphology under these conditions. Besides Hsp90, Cyps and FKBPs also play a role for intoxication of cells with DT because their specific pharmacological inhibitors CsA and FK506, respectively, delayed the DT-induced cell-rounding, too (Fig. 2b and Supplementary Fig. 3b). CsA or FK506 alone had no relevant effect on the morphology of the cells (Fig. 2b) and mock treatment did not interfere with the DT-induced cell-rounding (not shown).

These results were confirmed by analyzing DT-induced morphological alterations, measuring the incorporation of [^3^H]-leucine into newly synthesized proteins in CHO-K1 cells. As shown in Fig. 2c, pre-treatment with CsA protected CHO-K1 cells from cytotoxic DT-effects. The incubation of cells with DT resulted in a complete inhibition of protein synthesis, indicating the DT-catalyzed inactivation of EF-2. CsA-treatment rescued this DT-mediated effect in a concentration-dependent manner (Fig. 2c). Also, pre-treatment of cells with FK506 reduced the DT-catalyzed inhibition of protein synthesis in CHO-K1 cells (not shown). The results indicate that CsA as well as FK506 prevent the DT-mediated inhibition of protein synthesis by preventing the DTA-catalyzed inactivation of EF-2 in the cells.

A biochemical analysis of the ADP-ribosylation status of EF-2 from DT-treated HeLa cells further confirmed these results. As determined by sequential *in vitro* ADP-ribosylation of EF-2 from lysates of cells (Fig. 2d), less EF-2 was ADP-ribosylated in the cytosol of cells that were incubated with DT in the presence of either Rad, CsA or FK506, compared to cells treated with DT in absence of these inhibitors. In this assay, a weak signal in the Western blot indicates that less EF-2 was mono-ADP-ribosylated (i.e. biotinylated) during the *in vitro* enzyme reaction because most of the EF-2 was already mono-ADP-ribosylated by DT in the living cells and therefore did not serve as a substrate for the subsequent *in vitro* reaction. This result clearly indicates that less DTA activity was present in the cytosol of living cells when Hsp90, Cyps or FKBPs were inhibited. Based on the fact that Rad, CsA or FK506 did not inhibit DTA-catalyzed ADP-ribosylation of EF-2 *in vitro* (Fig. 2e), it is likely that the inhibitors interfere with the transport of DTA into the cytosol of DT-treated cells.

### The PPIase activity of intracellular Cyps is crucial for the cytotoxic effects mediated by DT

**Figure 3:**
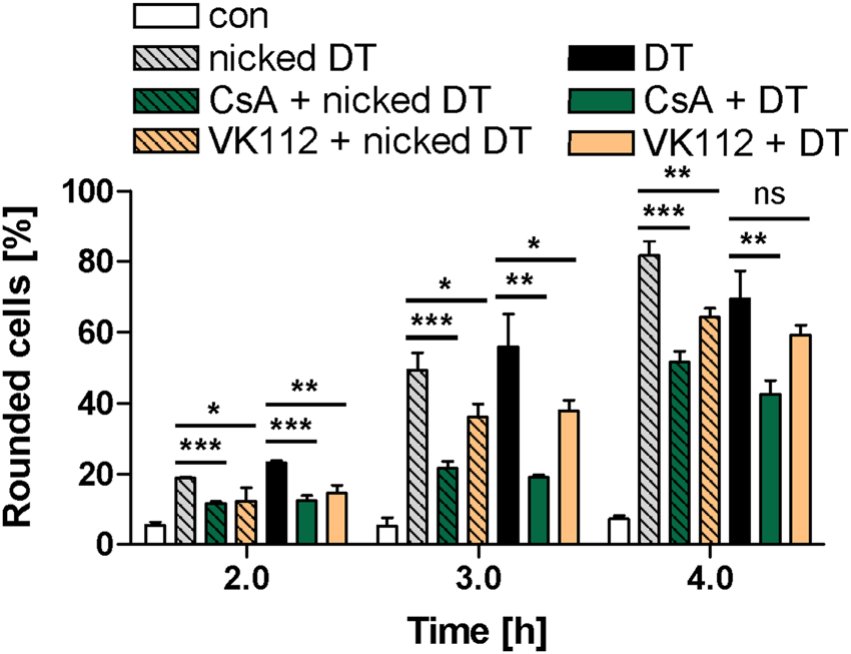
Effect of the non-immunosuppressive CsA-derivative VK112 on the intoxication of HeLa cells with DT. Cells were pre-incubated for 30 min at 37 °C with either CsA (20 µM) or VK112 (200 µM). Subsequently, DT (6.9 nM) or previously activated DT (nicked DT) (6.9 nM) was added and cells were incubated further. For control, cells were treated with DT in the absence of CsA or VK112, with the inhibitors alone or left untreated. After the indicated incubation periods, pictures were taken and the percentage of round cells was determined from the pictures to monitor the DT-intoxication over the time (mean ± SD; n = 3). Significance was tested between cells treated with the respective toxin in the absence (grey and black bars) and presence of CsA or VK112 (green and orange bars) using Student’s t-test (ns, not significant, *p < 0.05, **p < 0.01, ***p < 0.001).

Based on the observation that CsA-treatment protects cells from intoxication with DT, it was not possible to distinguish whether CsA mediates this effect via the inhibition of the PPIase activity of Cyps or via the complex formation between CsA-bound Cyp and calcineurin which triggers signal transduction independent of the PPIase activity^39^. Therefore, the CsA analogue VK112 was tested in direct comparison with CsA. VK112 is a tailored compound developed and characterized in our laboratories that specifically inhibits the PPIase activity of Cyps in cells, but does not mediate the Cyp/calcineurin complex formation and the resulting cellular responses^40^. Similar to CsA, treatment of cells with VK112 alone had no effect on the morphology of HeLa cells but delayed the intoxication of cells with DT as well as with DT which was proteolytically cleaved between its DTA and DTB domains *in vitro* to generate the activated (i. e. nicked DT (Fig. 3), as determined by monitoring the DT-induced cell-rounding. Nicked DT was included to demonstrate that VK112 has no effect on the proteolytic activation of DT on the surface of target cells or in endosomes but inhibits other steps.

In contrast, compound MM284, a tailored non-cell-permeable derivative of VK112, did not affect intoxication of HeLa cells with DT (Supplementary Fig. 4a), indicating that the inhibitory effect of VK112 towards an intoxication of cells with DT was mediated by intracellular Cyps while extracellular Cyps^41^ do not seem to play a role for this process. Thus, inhibition of the PPIase activity of intracellular Cyps mediates the protective effect against an intoxication of cells with DT. This is confirmed by microinjection of MM284 into the cytosol of HeLa cells which in this case also protected cells from DT-mediated cell-rounding (Supplementary Fig. 4b,c). As observed for CsA, VK112 did not inhibit the DTA-catalyzed ADP-ribosylation of EF-2 *in vitro* (Supplementary Fig. 4d) or the binding of DT to the cellular receptor (see Supplementary Fig. 6b). In conclusion, the results indicate that the PPIase activity of intracellular Cyps is crucial for the cytotoxic effects mediated by DT and suggest that Cyps play a role for the cellular uptake of the toxin.

### The PPIase activity of FKBP51 is crucial for the cytotoxic effects mediated by DT

FK506 inhibits the PPIase activity of both FKBP51 and FKBP52. In order to determine the relative contribution of FKBP51 and FKBP52 on intoxication of cells with DT, the effect of the FKBP51-selective inhibitor SAFit1^42^ on DT-intoxication was investigated. To this end, HeLa cells were pre-incubated with either FK506 or SAFit1, subsequently challenged with DT and the intoxication was monitored in terms of cell-rounding. As shown in Supplementary Fig. 5, less cell-rounding was detected in the presence of FK506 or SAFit1. Although both FKBP51 and FKBP52 bound to DTA *in vitro*, this result implicates that in living cells FKBP51 rather than FKBP52 is functionally involved in the cytotoxic effects mediated by DT.

### Hsp90 and the PPIases are required for the uptake of DTA into the cytosol of DT-treated cells

**Figure 4:**
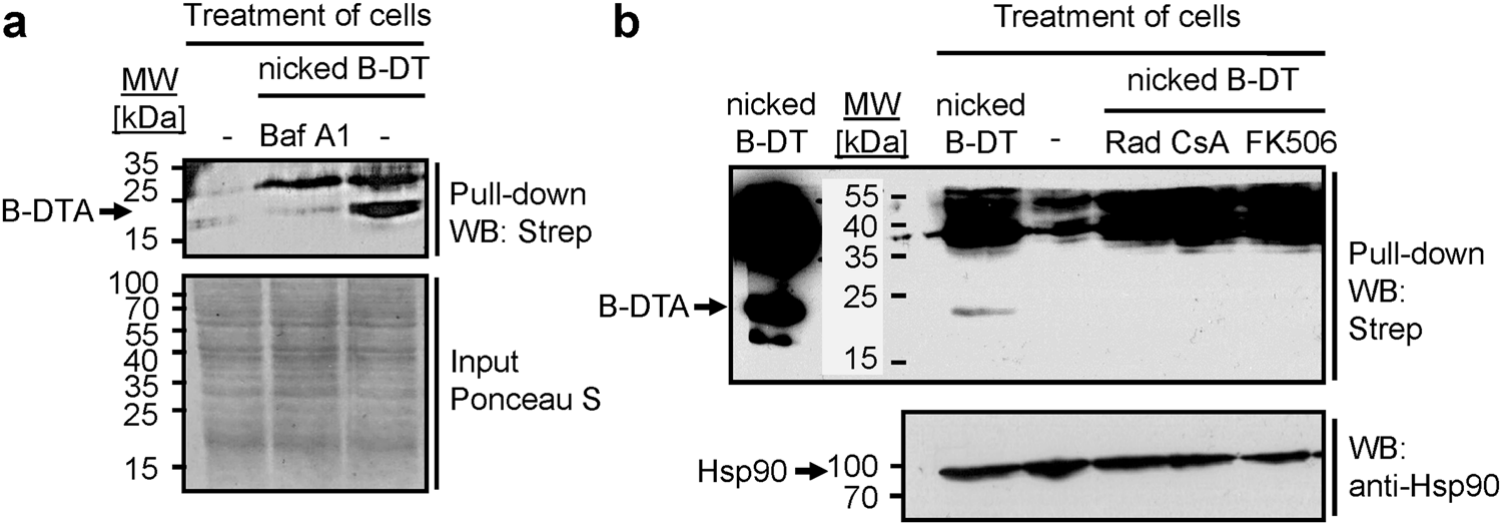
Effect of the pharmacological inhibition of Hsp90, Cyps and FKBPs on the uptake of DTA into the cytosol of HeLa cells treated with nicked biotin-labeled DT. (**a**) Detection of biotin-labeled DTA in the cytosol of HeLa cells. 10^6^ cells were suspended in 100 µl of serum-free medium and incubated for 1 h at 37 °C with 1 µg of nicked biotin-labeled DT in the presence or absence of Baf A1. For control, cells were left untreated. Subsequently, the cells were pelleted, washed and the cytosolic fractions were extracted by digitonin-treatment for subsequent pull-down of biotinylated proteins at 4 °C with streptavidin-agarose beads. The precipitated biotin-proteins were removed from the agarose beads by heating in SDS-sample buffer, separated by SDS-PAGE and detected by Western blotting with streptavidin-peroxidase (upper panel). To confirm that comparable protein amounts were used for the precipitation step, equal aliquots of the cytosolic fractions were removed prior to precipitation, subjected to SDS-PAGE, blotted onto nitrocellulose and stained with Ponceau S (lower panel). The Western blot panel was cropped for presentation purposes only. (**b**) Less DTA reaches the cytosol of DT-treated HeLa cells after pre-treatment of cells with Rad, CsA or FK506. HeLa cells were pre-incubated for 30 min at 37 °C with either Rad (20 µM), CsA (20 µM) or FK506 (10 µM) and then treated in suspension with nicked biotin-labeled DT. Biotinylated DTA was precipitated from digitonin-extracted cytosolic fractions and detected by Western blotting exactly as described in a. The upper panel shows the Western blot analysis of DTA (left lane, nicked B-DT as running control). Lower panel: Confirmation of comparable input of cytosolic proteins into the precipitation step by Western blotting against Hsp90. The Western blot panel was cropped for presentation purposes only.

To investigate the role of Hsp90 and the PPIases for the uptake of DTA into the cytosol of HeLa cells in more detail, biotin-labeled nicked DT (nicked B-DT) was used, which is essential for the direct investigation of the trans-membrane transport of DTA as described in more detail below. Nicked B-DT was biologically active and did not intoxicate Baf A1-treated cells (Supplementary Fig. 6a). As Baf A1, which prevents acidification of the endosomal lumen^43^, inhibits the translocation of DTA from endosomes into the cytosol, our results indicate that nicked B-DT behaves like unmodified DT regarding its cellular uptake. The uptake of B-DTA into the cytosol of nicked B-DT-treated cells was then further analyzed by Western blotting of the cytosolic fractions of these cells. To this end, cells were incubated with nicked B-DT in the absence or presence of Baf A1. The cytosolic fraction was then extracted by digitonin-treatment of these cells. B-DTA was subsequently precipitated from the cytosolic fractions using streptavidin-agarose and analyzed by Western blotting with the streptavidin-peroxidase detection system. As shown in Fig. 4a, a ~20 kDa protein was detected in the cytosol from nicked B-DT-treated cells, but not in the cytosol from untreated cells or from cells treated with nicked B-DT in the presence of Baf A1. This strongly suggests that the detected protein was B-DTA and further provides a proof-of-concept for the specific detection of translocated DTA by this approach. The higher migrating protein might be a contamination by biotinylated DT, may be a portion of DTB, because it did not appear in untreated cells. Noteworthy, in this approach, cells in suspension were used because of the large amount of cells which were necessary to detect B-DTA in the cytosolic fractions by Western blotting.

This assay was used to investigate whether treatment of cells with Rad, CsA or FK506 prior to DT application has an effect on the amount of B-DTA in the cytosol. As shown in Fig. 4b, no B-DTA was detected in the cytosol of nicked B-DT-treated HeLa cells in the presence of Rad, CsA or FK506 while B-DTA was detected in cells treated with nicked B-DT in the absence of an inhibitor. This result strongly suggests that the pharmacological inhibition of Hsp90, Cyps and FKBPs does not interfere with the proteolytic activation of DT during cellular uptake, as DT was already nicked *in vitro*, but inhibits the delivery of DTA into the host cytosol. Importantly, we confirmed that Rad, CsA and FK506 did not inhibit the binding of nicked B-DT to its receptor on HeLa cells (Supplementary Fig. 6b) which was shown to be specific, as demonstrated by pronase- or trypsin-treatment of cells prior to incubation with DT and by competition approaches for toxin binding and internalization (Supplementary Fig. 7a–d). The results suggest that the inhibitors interfere with a later step of DT uptake, such as the transport of DTA across endosomal membranes.

### Hsp90 and the PPIases facilitate the pH-induced transport of DTA across membranes into the cytosol of living cells

**Figure 5:**
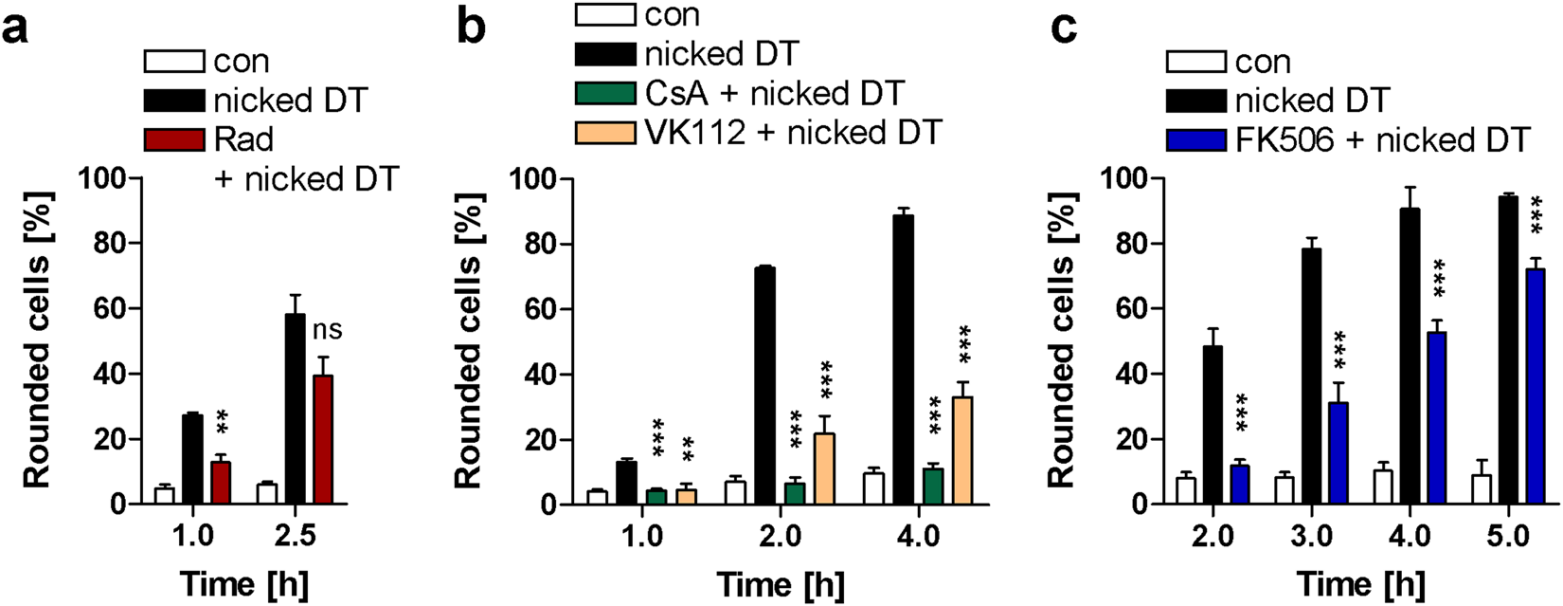
Effect of the pharmacological inhibition of Hsp90, Cyps and FKBPs on the pH-dependent translocation of DTA from cell-bound nicked DT across the cytoplasmic membrane of living HeLa cells. HeLa cells were pre-incubated for 30 min at 37 °C with 100 nM Baf A1 to block the normal uptake of DTA into the cytosol and then incubated with nicked DT (13.8 nM) for 10 min at 4 °C in serum-free medium to allow toxin-binding to the cell surface. Then, the medium was adjusted to pH 4.5 and the cells were incubated at 37 °C for further 15 min (pH-shift). For control, cells were incubated for 15 min with neutral medium instead of the pH-shift. Subsequently, the cells were further incubated in warm and neutral serum-containing medium containing Baf A1. Effect of a pre-incubation of cells with either Rad (10 µM) (**a**), CsA (20 µM) or VK112 (200 µM) (**b**), or FK506 (10 µM) (**c**) on the pH-dependent translocation of cell-bound nicked DT. After the indicated incubation periods, pictures were taken and the percentage of rounded cells was determined. Values are given as mean ± SD (n = 3); significance was tested between cells treated with nicked DT in the absence (black bars) and presence of the respective inhibitor (colored bars) using Student’s t-test (ns, not significant, **p < 0.01, ***p < 0.001).

**Figure 6:**
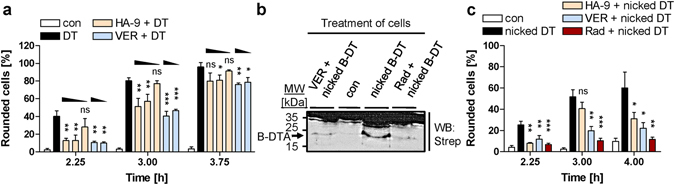
Hsp70 is crucial for the pH-dependent transport of DTA across cell membranes into the host cell cytosol. (**a**) Effect of VER155008 and HA-9 on intoxication of HeLa cells with DT. Cells were pre-incubated for 30 min at 37 °C with HA-9 (10, 20, 50 µM) or VER155008 (VER, 10, 50 µM) or left untreated. Then, DT (6.9 nM) was added and cells were further incubated with DT alone (DT) or with DT and each inhibitor. For control, cells were left untreated (con). After the indicated times, pictures were taken to determine the percentage of rounded cells. Values are mean ± SD (n = 3); significance was tested between cells treated with DT in the absence and presence of inhibitor using Student’s t-test (ns, not significant, *p < 0.05, **p < 0.01, ***p < 0.001). (**b**) Less DTA reaches the cytosol of DT-treated HeLa cells pre-treated with VER155008. HeLa cells were pre-incubated for 30 min at 37 °C with either VER155008 (VER, 20 µM), or for control Rad (20 µM). Then, the cells were treated in serum-free medium with nicked biotin-DT (nicked B-DT). For control, cells were incubated with nicked B-DT without inhibitor (nicked B-DT) or left untreated (con). Biotin-DTA from digitonin-extracted cytosolic fractions was detected by Western blotting with streptavidin-peroxidase (Strep) (upper panel). The Western blot panel was cropped for presentation purposes only. The comparable input of cytosolic proteins into the analysis was confirmed by Ponceau S staining (not shown). (**c**) Effect of VER155008 and HA-9 on the pH-dependent translocation of DTA from cell-bound nicked DT across the cytoplasmic membrane. All cells were pre-incubated for 30 min at 37 °C with 100 nM Baf A1 to block normal uptake of DTA into the cytosol. In addition, cells were treated for 30 min with HA-9 (20 µM), VER155008 (VER, 20 µM) or Rad (20 µM) and incubated in serum-free medium for 15 min at 4 °C with nicked DT (13.8 nM) to allow binding. For control, cells were treated with Baf A1 (con) or with Baf A1 plus nicked DT (nicked DT). Subsequently, all cells were exposed for 10 min at 37 °C to pH 4.5 and further incubated (37 °C, neutral medium, Baf A1). After the indicated times, pictures were taken to determine the percentage of rounded cells. Values are mean ± SD (n = 3); significance was tested between cells treated with nicked DT in the absence and presence of inhibitor using Student’s t-test (ns, not significant, *p < 0.05, **p < 0.01, ***p < 0.001).

The exposure of cell-bound nicked DT to acidic medium induces pore-formation by DTB in cytoplasmic membranes and translocation of DTA through DTB pores across cytoplasmic membranes into the cytosol in the presence of Baf A1, which allows to investigate the membrane transport of DTA in an isolated manner independent from the other steps of DT uptake^6, 15, 23, 44, 45^. To test whether Hsp90, Cyps and FKBPs facilitate the pH-driven transport of DTA across membranes in living cells, we performed this well-established assay to investigate the translocation of DTA from cell-bound nicked DT directly across the cytoplasmic membrane after an acidic pulse. Nicked DT was used because the proteolytic activation of native DT by the cells does not efficiently occur under conditions where DT is bound to cells at 4 °C and is not taken up via endosomes where the DT proteolysis occurs normally (Supplementary Fig. 8). HeLa cells were either pre-treated with Rad, CsA or FK506, or left untreated for control. Importantly, all cells were treated with Baf A1 to inhibit the normal uptake of DT via acidic endosomes. Subsequently, the cells were incubated at 4 °C with nicked DT to enable its binding to the cell receptor and translocation of the DTA into the cytosol was triggered by an acidic pulse. DTA translocation into the cytosol was monitored by analyzing the DTA-catalyzed cell-rounding. The results shown in Supplementary Fig. 8 clearly indicate that DTA translocation only occurred under acidic conditions, confirming the specificity of this toxin translocation assay. Importantly, pre-treatment of cells with either Rad (Fig. 5a, see also Fig. 6c), CsA, VK112 (Fig. 5b), or FK506 (Fig. 5c) prior to the application of nicked DT clearly delayed the DT-induced cell-rounding.

This result implicates that Hsp90, Cyps and FKBPs are crucial for the pH-driven translocation of DTA across cytoplasmic membranes in living cells. Since this assay closely mimics the situation of acidified endosomal vesicles in living cells, the findings strongly suggest that the chaperone activity of Hsp90 and the PPIase activities of Cyps and FKBPs, facilitate the translocation of DTA from acidic endosomes into the cytosol during the normal DT uptake into human cells.

### Hsp70 facilitates the transport of DTA across cell membranes into the cytosol

To investigate whether Hsp70 and/or Hsc70, which we both identified as novel binding partners of DTA, also play a role for the mode of action of DT, HeLa cells were pre-treated with the specific inhibitors VER155008 and HA-9, challenged with DT and the DT-induced cell-rounding was monitored over the time. VER155008 binds to the ATP binding pocket of Hsc70 and Hsp70 thereby inhibiting their foldase activity^46^. In contrast, HA-9 specifically binds to the peptide-binding site of Hsp70^47^. For the Hsp70 chaperone DnaK it was shown earlier that the amidic peptide bond *cis/trans* isomerase (APIase) activity catalyzing the *cis/trans* isomerization of non-proline bonds in protein resides in the peptide binding pocket^48^.

As shown in Fig. 6a, both inhibitors delayed the DT-induced cell-rounding. The finding that VER155008 slows down intoxication with DT suggests that the chaperone activity of Hsp70 and/or Hsc70 are involved in the cytotoxic mode of action of DT. However, the observation that HA-9 also inhibits the DT effects on cells suggests that the APIase activity of Hsp70 is involved in the intoxication of cells with DT and implicates that Hsp70 rather than Hsc70 might be crucial for the mode of action of DT. The Hsc70/Hsp70 inhibitors had no effect on the enzymatic activity of DT, as tested by ADP-ribosylation of EF-2 (Supplementary Fig. 9a), or binding of DT to HeLa cells (Supplementary Fig. 9b). However, less DTA protein was detected in the cytosol of DT-treated cells, if these cells were pre-incubated with VER155008, comparable to a pre-incubation with the Hsp90 inhibitor Rad (Fig. 6b). Both, VER155008 and HA-9 inhibited the pH-driven transport of the DTA moiety from cell-bound nicked DT across the cytoplasmic membranes of HeLa cells (Fig. 6c), suggesting that the chaperone activity and the APIase activity of Hsp70 are crucial to facilitate the translocation of DTA across the membranes of acidified endosomal vesicles during the normal uptake of DT in human cells. Besides these pharmacological approaches, the involvement of Hsp70 in DT uptake was further underlined by RNAi-mediated Hsp70-depletion in HeLa cells, which also resulted in a delay of DT-intoxication as demonstrated by a reduced number of rounded cells (Supplementary Fig. 10a) and a diminished amount of ADP-ribosylated EF-2 in their cytosol (Supplementary Fig. 10b).

The involvement of extracellular Hsp70 was excluded by using the non-cell-permeable Hsp70 inhibiting peptide NRLLLTG that showed no effect on the intoxication of HeLa cells with DT in contrast to the cell-permeable Hsp70 inhibitor VER155008 (Supplementary Fig. 9c).

In conclusion, the results implicate that Hsp70 facilitates the same step of DT uptake into cells as Hsp90 and the PPIases. Of note, this is the first report that the chaperone as well as the APIase activities of Hsp70 are involved in the intracellular membrane transport of the enzyme domain of DT.

## Discussion

By performing a series of biophysical, biochemical and pharmacological approaches, we were able to show that the components of the Hsp90 machinery including Hsp90, Hsp70, Cyp40 and FKBP51 bind to and facilitate the pH-dependent membrane transport of DTA during the uptake of native DT into human cells. The first direct evidence that host cell factors play a crucial role for the transport of DTA from the lumen of acidic endosomes into the cytosol *in vitro* was provided by Lemichez *et al*., who established an *in vitro* assay and demonstrated that translocation of DTA from DT-loaded purified endosomes requires the addition of cytosolic factors including the coatomer protein ß-COP^18^. Later, Ratts and co-workers used this assay to demonstrate that Hsp90 and thioredoxin reductase 1 (TrR-1) are components of a cytosolic translocation factor complex that binds to DTA on the cytosolic side of the endosomal membrane and facilitates translocation of DTA from the endosomal lumen, which was pre-loaded with a DTA fusion toxin^29^. Prompted by these results, we then showed that Hsp90 and CypA interact with DTA and facilitate the transport of the recombinant LF_N_DTA fusion toxin through the PA63 pore of the anthrax toxin across endosomal membranes *in vitro* and in cells^30^.

In the present study, we investigated for the first time the role of host cell chaperones for the uptake of wild-type DT into living human cells. In summary, the results revealed that Hsp90, Hsp70 as well as PPIases of the Cyp and FKBP families are involved in the pH-dependent transport of the enzyme domain of internalized DT across membranes into the cytosol. In addition to the already known DTA binding partners Hsp90 and CypA, we identified the PPIases Cyp40 and FKBP51/FKBP52 as well as Hsp70/Hsc70 as novel binding partners of DTA *in vitro* and demonstrated their specific and direct binding to DTA by different biophysical and biochemical approaches. These results confirm and extend earlier findings by Ratts *et al*.^29^ and our group^30^ that Hsp90 and CypA bind to the DTA and facilitate the membrane transport of DTA-containing fusion toxins *in vitro* and in living cells and further verify the hypothesis that host cell chaperones assist internalized DT to deliver its enzyme domain from acidified endosomes into the cytosol.

**Figure 7:**
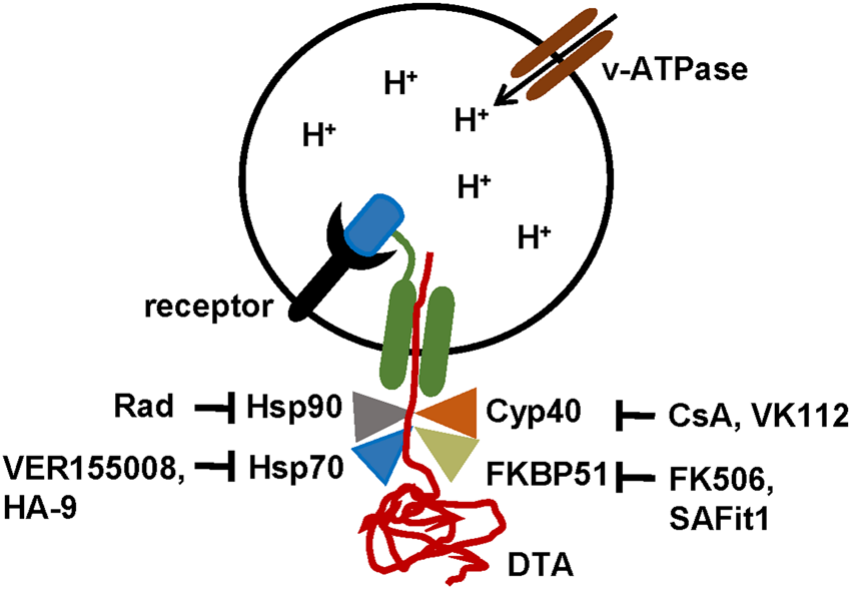
Schematic model of the role of Hsp90, Hsp70 and the PPIases of the Cyp and FKBP families during the transport of DTA across the endosomal membrane into the cytosol (modified from ref. 1). Blue, R-domain of DT; green, T-domain of DT. Detailed explanations are given in the text. Rad, radicicol; Cyp, cyclophilin; CsA, cyclosporine A; DTA, enzyme domain of diphtheria toxin; FKBP, FK506 binding protein; Hsp, heat shock protein; VK112, specific cyclophilin inhibitor (explained in text); FK506, FKBP inhibitor; SAFit1, specific FKBP51 inhibitor; VER155008, inhibitor of Hsp70 and Hsc70; HA9, specific Hsp70 inhibitor.

The use of the inhibitors Rad, VER155008, CsA and FK506 revealed that Hsp90, Hsp70/Hsc70, Cyps and FKBPs, respectively, facilitate the pH-dependent transport of DTA across membranes of living cells into their cytosol when the cells were exposed to wild-type DT. Treatment of cells with each inhibitor prior to DT application prevented the DTA-catalyzed ADP-ribosylation of EF-2 in the cytosol, thereby protecting cells from intoxication. However, treatment with the inhibitors led to a temporal delay of intoxication rather than a complete inhibition, which is plausible given the extremely potent mode of action of DT where only few molecules of the DTA in the cytosol might be sufficient to kill a cell^49^. None of the compounds inhibited the enzyme activity of DTA, binding of DT to cells or the nicking of DT in the medium and/or on cells. Importantly, only negligible amounts of DTA reached the cytosol of DT-treated cells in the presence of each inhibitor and the pH-dependent membrane transport of DTA was prevented when the chaperones/PPIases were pharmacologically inhibited, implicating that each of the identified host cell factors is involved in the membrane transport and/or refolding of DTA (Fig. 7). The observed better protection of the cells against the nicked DT compared to DT might be due to the more synchronous cytosolic delivery of DTA from nicked DT, which does not require the proteolytic activation on and/or in the cells.

Additional experiments with more specific pharmacological inhibitors, developed and characterized in our laboratories, implicated that Hsp70 and FKBP51 play a role for the uptake of DTA into the cytosol and provided further insight into the molecular mode of action underlying the interaction of the host cell factors with DTA. By using non-cell-permeable inhibitors, it was confirmed that the intracellular chaperones/PPIases facilitate DTA uptake. Application of the non-immunosuppressive CsA-derivative VK112^50^ revealed that the PPIase activity of Cyps, and not their interaction with calcineurin, is crucial for membrane transport of DTA in cells and the use of selective FKBP inhibitors identified FKBP51 as the essential FKBP for this step.

Moreover, the use of our tailored cell-permeable Hsp70-specific inhibitor HA-9^47^ in addition to the Hsp70/Hsc70-specific inhibitor VER155008, was an essential prerequisite to demonstrate for the first time that Hsp70 plays a role in cellular uptake of DT by facilitating the trans-membrane transport of DTA in cells.

Interestingly, Hsp70, Cyp40 and FKBP51 are known co-chaperones of Hsp90 which bind to Hsp90 in a timely concerted manner and are important components of the Hsp90 machinery in the regulation of client proteins such as the steroid hormone receptor^37, 51, 52^. By performing *in vitro* translocation studies with isolated early endosomes, Ratts and co-workers identified Hsp90 as part of a cytosolic translocation factor complex, which binds to the DTA domain in a DTA-containing fusion toxin and facilitated its transport from the endosomal lumen across endosomal membranes into the cytosolic fraction^29^. Although Cyps and FKBPs have not been identified as components of the translocation factor complex that interacts with DTA *in vitro* so far, cyclophilin was found in complexes from yeast that contain the further known cytosolic factors required for DTA translocation^53^.

The interaction of Hsp90 and other factors identified in this study with the ADP-ribosyltransferase domain to facilitate its pH-dependent membrane transport in cells is very likely a specific, common and unique feature of bacterial ADP-ribosylating toxins. Besides DTA^29, 30^, this functional interaction was demonstrated for all members of the family of binary actin ADP-ribosylating toxins^47, 50, 54–58^, for the actin ADP-ribosylating PTC3 toxin of *Photorhabdus luminescens* ^59^ and for Hsp90 also for cholera toxin^60^. Moreover, we demonstrated that an isolated ADP-ribosyltransferase domain directly interacts with and depends on the activities of Hsp90, Cyps and FKBPs for its membrane translocation^59^. Toxins, which are not ADP-ribosyltransferases but exploit comparable cellular uptake pathways via acidic endosomal vesicles and translocation subunits, such as the binary anthrax toxins or the large cytotoxins A and B from *Clostridium difficile*, do not require these factors for cellular uptake of their enzyme subunits^54, 57^. In line with the results obtained for DTA in this study, we recently found that the enzyme components of clostridial binary actin ADP-ribosylating toxins C2 and iota directly bind to Hsp70 *in vitro* and that VER155008 inhibits their pH-dependent membrane translocation in cells, implicating a novel common role for Hsp70 in the membrane transport of bacterial ADP-ribosyltransferases^47^.

Finally, the results not only provide new insights into the molecular mechanisms underlying the uptake of DT into human cells but might also be of medical interest^61^. The newly identified host cell factors represent novel drug targets to prevent intoxication with DT, even when DT is already internalized into cells, because their targeted pharmacological inhibition in human cells prevents the uptake of DTA into the cytosol. We recently demonstrated that the tailored non-immunosuppressive CsA-derivative VK112 efficiently protected cells from intoxication with binary actin ADP-ribosylating toxins from pathogenic clostridia^50^ by inhibiting the intracellular membrane transport of the enzyme components of these toxins into the host cell cytosol. Therefore, such compounds might represent lead compounds for the development of novel pharmacological inhibitors against diphtheria. Although there is an efficient vaccination against diphtheria toxin in many countries, this serious childhood disease is still endemic in some regions of the world and a public health problem in many developing countries. Moreover, since 1990, diphtheria cases in adults were increasing in several European countries due to a lack of immunity^62^. In addition to diphtheria antitoxin, which must be applied rapidly to bind the extracellular DT prior to internalization into cells, the novel chaperone/PPIase inhibitors, which prevent the transport of DTA from already internalized DT into the host cell cytosol, could be a valuable addition to the therapeutic strategies to prevent and treat diphtheria.

## Materials and Methods

### Materials

MEM cell culture medium and fetal calf serum (FCS) were from Invitrogen (Karlsruhe, Germany) and materials for cell culture from TPP (Trasadingen, Switzerland). Complete^®^ protease inhibitor, streptavidin-peroxidase and pronase were from Roche (Mannheim, Germany), protein weight marker PageRuler Prestained Protein Ladder^®^ from Thermo Scientific (Bonn, Germany), biotin-NAD^+^ from R&D Systems GmbH (Wiesbaden-Nordenstadt, Germany) and trypsin and trypsin inhibitor form Sigma-Aldrich (Deisenhofen, Germany). Bafilomycin (Baf) A1 was from Calbiochem (Bad Soden, Germany), radicicol (Rad), FK506 from Sigma (Steinheim, Germany) and CsA from Fluka (Munich, Germany). The enhanced chemiluminescence (ECL) system was from Millipore (Schwalbach, Germany), streptavidin-agarose beads from Pierce (Bonn, Germany), diphtheria toxin (DT) and non-toxic DT-mutant CRM197 from Calbiochem/Merck KGaA (Bad Soden/Darmstadt, Germany) and talon^®^ CellThru Resin from Clontech (Heidelberg, Germany). VER155008 was from Tocris Bioscience (Wiesbaden-Nordenstadt, Germany). Synthesis of the CsA derivatives MM284 and VK112^40, 63^ and of HA-9 was performed as described^64^. The antibody against Hsp70 was from Enzo Life Sciences (Lörrach, Germany), against Hsp90 from Santa Cruz Biotechnology (Heidelberg, Germany) and against Cyp40 and actin from Thermo Scientific (Bonn, Germany). The secondary peroxidase-coupled anti-rabbit and anti-mouse antibodies were from Santa Cruz Biotechnology (Heidelberg, Germany). SAFit1^42, 65^ was synthesized as described. The peptide NRLLLTG was synthesized using standard solid-phase peptide synthesis methods.

### Protein expression and purification

The following recombinant proteins were produced as described earlier: DTA^31^, CypA^66^, human Hsp90β^67^, FKBP12, Cyp40, FKBP51, FKBP52, Hsp70 and Hsc70^47, 50^ and the FK1 domains of FKBP51^68^ and FKBP52^69^.

### Cell culture and cytotoxicity assays

HeLa cells were cultivated at 37 °C and 5% CO_2_ in MEM containing 10% heat-inactivated FCS, 1.5 g/l sodium bicarbonate, 1 mM sodium pyruvate, 2 mM L-glutamine, 0.1 mM non-essential amino acids and 1% penicillin-streptomycin. Cells were trypsinized and reseeded for at most 30 times. For cytotoxicity experiments, cells were seeded in culture dishes and incubated in serum-free medium with DT. To inhibit the PPIase activity of Cyps, the cells were incubated for 30 min with the indicated concentrations of either CsA or the non-immunosuppressive CsA derivative VK112^50^. Extracellular Cyps were inhibited by 30 min incubation of cells with the non-cell-permeable derivative MM284^50^. To inhibit the PPIase activity of FKBPs, the cells were incubated for 30 min with the indicated concentrations of FK506. For the selective inhibition of FKBP51, cells were incubated for 30 min with SAFit1^42^. To inhibit the chaperone activity of Hsp90, cells were incubated for 30 min with Rad^54^. To inhibit Hsp70 activity, cells were incubated for 30 min with the indicated concentrations of VER155008 or HA-9^47^. To inhibit the activity of extracellular Hsp70, cells were incubated for 30 min with the non-cell-permeable peptide NRLLLTG^47^. Afterwards, DT was added and cells were further incubated at 37 °C with DT plus inhibitor. According to the indicated incubation periods, the DT-mediated cell-rounding as specific endpoint of the intoxication process was visualized by using a Zeiss Axiovert 40 CFL microscope (Oberkochen, Germany) with a Jenoptik progress C10 CCD camera (Jena, Germany). The percentage of round, i.e. intoxicated, cells was assigned from the pictures.

### Measurement of protein biosynthesis in CHO-K1 cells by incorporation of [3 H]-leucine

Incorporation of [^3^H]-leucin into CHO-K1 cells was performed as described^30^. In brief, 30,000 cells/well were pre-treated with or without increasing concentrations of the indicated inhibitor for 30 min at 37 °C, DT (3.4 nM) was added and the cells were further incubated for 2 h at 37 °C. Then, the medium was replaced with L-leucine-free medium supplemented with [^3^H]-leucine and after an additional hour of incubation at 37 °C, cells were washed three times with cold PBS. Cells were lysed with 0.1% SDS and proteins precipitated by 10% trichloroacetic acid. The amount of incorporated [^3^H]-leucine was measured by scintillation counting.

### SDS-PAGE and immunoblot analysis

For immunoblot analysis, samples were denatured in reducing sample buffer at 95 °C and subjected to SDS-PAGE^70^. Afterwards, the proteins were transferred to a nitrocellulose membrane (Whatman, Dassel, Germany) and the membrane was blocked for 1 h with 5% non-fat dry milk in phosphate-buffered saline (PBS) containing 0.1% Tween-20 (PBST). The biotin-labeled proteins (DTA, DTB or EF-2) were detected by using streptavidin-peroxidase. For detection of precipitated proteins, the nitrocellulose membrane was probed with specific antibodies against Hsp70, Hsp90 or Cyp40, followed by incubation with a secondary peroxidase-coupled antibody. In all cases, proteins were visualized by using the ECL system according to the manufacturer’s instructions.

### Proteolytic activation (Nicking) of DT

For *in vitro* activation^7^, intact DT was treated with trypsin (3 µg/ml) for 1 h at 37 °C and then kept on ice. Thereafter, the complete separation of DTA and DTB was tested by reducing SDS-PAGE followed by Coomassie staining. If DTA and DTB were completely separated, trypsin was neutralized by incubation with trypsin inhibitor (30 µg/ml, 80 min, 4 °C) and the concentration of nicked DT was determined by SDS-PAGE.

### Biotin-labeling of DT and DTA

For biotin-labeling, DT and DTA were incubated with EZ-Link sulfo-NHS-Biotin (Pierce) for 2 h at 4 °C according to the manufacturer’s instructions and free sulfo-NHS-Biotin was removed by using Micro Bio-Spin^TM^ Chromatography Columns from Bio-Rad (Munich, Germany). The biological activities of biotinylated DT and DTA were confirmed by cytotoxicity assay and *in vitro* ADP-ribosylation of EF-2, respectively.

### ADP-ribosylation of EF-2 by DTA in a cell free system

HeLa lysate (10 µg of protein) was incubated for 30 min at 37 °C with Rad (20 µM), CsA (20 µM), VK112 (200 µM), FK506 (10 µM), HA-9 (20 µM) or VER155008 (20 µM). Thereupon, DTA (100 ng) and biotin-labeled NAD^+^ (10 µM) were added and samples were incubated for 10 or 30 min (as indicated in the respective experiment) at 37 °C. Subsequently, the samples were subjected to SDS-PAGE, blotted onto a nitrocellulose membrane and the ADP-ribosylated, i.e. biotin-labeled, EF-2 was detected using streptavidin-peroxidase and the ECL system. For comparison of the samples, the intensity of biotin-labeled EF-2 was evaluated via densitometry by using the Adobe Photoshop software (version 7.0, Adobe Systems GmbH, Munich, Germany).

### Toxin translocation assay with intact cells

The pH-dependent translocation of DT across the cytoplasmic membranes of living HeLa cells was performed as described^6, 44^. In brief, HeLa cells were pre-incubated in serum-free medium with Baf A1 (100 nM) plus one of the inhibitors CsA (20 µM), VK112 (200 µM), FK506 (10 µM), Rad (10 µM or 20 µM, as indicated in the respective experiment), HA-9 (20 µM) or VER155008 (20 µM) for 30 min at 37 °C. Then, the cells were incubated at 4 °C with nicked DT (13.8 nM) to allow binding of DT to the cell surface. Subsequently, the cells were exposed to an acidic pulse (pH 4.5) for 10 or 15 min (as indicated in the respective experiment) at 37 °C to trigger pH-driven membrane insertion of DTB and translocation of DTA across the membranes into the host cell cytosol. Thereafter, the cells were further incubated at 37 °C in neutral medium containing FCS and Baf A1. The DTA-induced cell-rounding as specific endpoint for DTA translocation was monitored by photography and from the pictures, the percentage of round cells was determined.

### Dot blot analysis of the binding between DTA and immobilized host cell factors

A serial dilution (2 µg, 1 µg, 500 ng, 250 ng or 1 µg, 500 ng, 250 ng, as indicated in the respective experiment) of each of the recombinant proteins Hsp90, Hsp70, Hsc70, CypA, Cyp40, FKBP12, FKBP51, FKBP52, FKBP51FK1, FKBP52FK1 and C3bot was immobilized onto a nitrocellulose membrane by vacuum aspiration using the Bio-Rad dot blot system according to the manufacturer’s instructions. Ponceau S staining of the membrane was performed, followed by blocking of the membrane with 5% non-fat dry milk in PBST. After this, the membrane was cut and an overlay with biotin-DTA (9.5 nM) or PBST for control was performed for 1 h. After extensive washing, bound biotin-DTA was detected with streptavidin-peroxidase by using the ECL system. For comparison of the binding of native vs. denatured biotin-DTA to the chaperones and PPIases, DTA was either treated with PBS or 6 M guanidine hydrochloride (1 h, RT) before applying to the dot blot analysis, as described earlier for clostridial binary toxins^50^.

### Identification of cellular binding partners of His_6_-DTA by pull-down

HeLa lysate (1,500 µg of protein) was incubated with His_6_-DTA (4 µg or 8 µg) or without His_6_-DTA (control) for 30 min at RT. Thereupon, the lysates were adjusted to a volume of 1 ml with PBS, then added to a 30-µl-volume bed (1:1 in PBS) of Talon^®^ CellThru beads and incubated for 1 h at 4 °C overhead. Before addition to the beads, an aliquot (10 µl) of each probe was taken as input control. The co-precipitated proteins were detected by Western blotting.

### Analysis of cytosolic DTA from digitonin extracted cells

To detect the amount of DTA that entered the cytosol after treatment of cells with DT, 10^6^ HeLa cells were suspended in 100 µl of serum-free medium and incubated for 1 h at 37 °C with 1 µg of nicked biotin-labeled DT. The cells were pelleted, washed and their cytosolic fractions extracted by digitonin-treatment as described previously^50^. The cytosolic fractions were incubated at 4 °C with streptavidin-agarose beads and the beads were pelleted and washed three times with PBS. The precipitated biotin-DTA was removed by heating the beads in SDS-sample buffer and detected by Western blotting with streptavidin-peroxidase. To confirm the protein transfer onto the membrane, the blotted proteins were stained with Ponceau S. To test whether inhibition of the host cell chaperones and PPIases interferes with the amount of translocated DTA, cells were incubated for 30 min with the indicated concentrations of each inhibitor prior to the application of biotin-DT and the cytosolic portion of biotin-DTA was analyzed exactly as described before.

### Reproducibility of experiments and statistics

All experiments were performed independently at least two times and results from representative experiments are shown in the figures. Values (n = 3) are calculated as mean ± standard deviation (SD) using the Prism4 Software (GraphPad Software, La Jolla, USA). Significance was tested using Student’s t-test as indicated in detail in the respective figure legends. Where cropped Western blots are displayed, the blot panels were cut and recombined for presentation purposes only. All corresponding protein bands were originally detected on the same membrane and X-ray film.

## Electronic supplementary material

Supplementary Information
