# Supplementary material for: The Hsp90 machinery facilitates the transport of diphtheria toxin into human cells
Source: Sci Rep. 2017 Apr 4;7:613. doi: 10.1038/s41598-017-00780-x (PMC5429619; doi:10.1038/s41598-017-00780-x)
Supplement: Supplementary file 1 — Supplementary Information [file 41598_2017_780_MOESM1_ESM.doc]

**The Hsp90 machinery facilitates the transport of diphtheria toxin into human cells**

Manuel Schuster^1^*, Leonie Schnell^1^*, Peter Feigl^1^*, Carina Birkhofer^1^, Katharina Mohr^1^, Maurice Roeder^1^, Stefan Carle^1^, Simon Langer^1^, Franziska Tippel^2^, Johannes Buchner^2^, Gunter Fischer^3^, Felix Hausch^4,5^, Manfred Frick^6^, Carsten Schwan^7^, Klaus Aktories^7^, Cordelia Schiene-Fischer^8^, Holger Barth^1§^

^1^Institute of Pharmacology and Toxicology, University of Ulm Medical Center, Ulm, Germany

^2^Munich Center for Integrated Protein Science and Department Chemistry, Technical University of Munich, Munich, Germany

^3^Max Planck Research Unit for Enzymology of Protein Folding Halle, Halle (Saale), Germany

^4^Institute for Organic Chemistry and Biochemistry, Technical University Darmstadt, Darmstadt, Germany

^5^Department of Translational Research in Psychiatry, Max Planck Institute of Psychiatry, Munich, Germany

^6^Institute of General Physiology, University of Ulm, Ulm, Germany

^7^Institute of Experimental and Clinical Pharmacology and Toxicology, University of Freiburg, 79104 Freiburg, Germany

^8^Institute for Biochemistry and Biotechnology, Martin Luther University Halle-Wittenberg, Halle (Saale), Germany

**Running Title:** Hsp90, Hsp70, cyclophilins and FKBPs facilitate uptake of diphtheria toxin in human cells

*contributed equally to this work

^§^Correspondent footnote:

Dr. Holger Barth

Institute of Pharmacology and Toxicology, University of Ulm Medical Center

Albert-Einstein-Allee 11, D-89081 Ulm, Germany

Tel.: 49-731-50065503, Fax: 49-731-50065502, e-mail : holger.barth@uni-ulm.de

**Supplementary Methods**

*Sequential ADP-ribosylation of EF-2 in lysates from DT-treated cells*

After incubation with DT in the presence or absence of the inhibitors Rad (20 µM), CsA (20 µM) or FK506 (10 µM), as well as after treatment of Hsp70-depleted cells with or without DT, cells were lysed and scraped off in ADP-ribosylation-buffer containing complete® protease inhibitor. Lysates (20 µl) were incubated for 30 min with biotin-NAD^+^ (10 µM) and DTA (300 ng or 100 ng, as indicated in the respective experiment). SDS-PAGE and Western Blot analysis were performed and the biotinylated (i.e. ADP-ribosylated) proteins were detected using streptavidin-peroxidase and the ECL system.

*Analysis of the binding of biotin-labeled DT to the cell surface*

HeLa cells were pre-incubated for 30 min at 37 °C in serum-free medium with either Rad (20 µM), CsA (20 µM), VK112 (200 µM), FK506 (10 µM), HA-9 (20 µM) or VER155008 (20 µM). Subsequently, cells were incubated for 15 min at 4 °C with nicked, biotin-labeled DT (6.9 nM or 13.8 nM, as indicated in the respective experiment) to enable binding of the toxin to the cell surface. Thereafter, cells were washed twice with cold PBS, scraped off, lysed and equal amounts of lysate protein were subjected to SDS-PAGE and blotted onto nitrocellulose. The biotin-labeled DT was detected by Western blotting with streptavidin-peroxidase and the ECL system. The intensity of biotin-DTB was evaluated via densitometry to estimate the amount of bound toxin. To confirm the specific binding of biotin-labeled DT to its cell surface receptor, HeLa cells were incubated for 30 min at 37 °C with pronase (500 µg/ml) or trypsin (100 µg/ml) to remove cell surface proteins. The activities of pronase or trypsin were then inhibited by addition of serum (10 %) or trypsin inhibitor (1000 µg/ml), respectively. Subsequently, cells were washed three times with serum-containing medium prior to incubation with biotin-labeled DT (34.5 nM) for 10 min at 4 °C. Thereafter, the supernatant of each sample was collected and cells were washed three times with cold PBS and analyzed by Western blotting for the amount of bound biotin-DT. In addition, Western blot analysis of the supernatant for the presence of biotin-DT was performed using streptavidin-peroxidase and the ECL system to exclude the digestion of biotin-DT by proteases.

*Competition assays of DT and CRM197 for binding and cellular uptake*

To evaluate the specific binding of DT to its cell surface receptor HB-EGF, two competition assays with the enzymatically inactive DT mutant CRM197 were performed. To evaluate the competition for cellular uptake, HeLa cells were incubated with DT (17.25 nM) alone and in combination with an excess of CRM197 (172.5 nM) at 37 °C. To determine the competition for cell surface binding, intact HeLa cells were treated with nicked DT (17.25 nM) alone and in combination with a tenfold excess of CRM197 (172.5 nM) for 30 min at 4 °C to allow toxin-binding. Subsequently, the medium was exchanged and cells were further incubated at 37 °C. Cellular uptake of DT was evaluated by monitoring DT-mediated cell-rounding via phase contrast microscopy.

*Isothermal titration calorimetry*

ITC experiments were performed using a VP-ITC (MicroCal, Munich, Germany). Prior to the experiment, all buffers were filtered through filter membranes with a pore size of 0.2 µm (Whatman, Dassel, Germany) and degassed. Protein solutions were dialyzed against the assay buffer (35 mM HEPES, pH 7.8). In a typical experiment, 300 μl of a 180 µM or a 100 µM solution of Cyp40, FKBP51, FKBP52, CypA or FKBP12 were titrated in 15 µl-steps to a 10 µM DTA solution, respectively. Analysis of the Hsp70/DTA interaction was performed by titration of a 190 µM solution of DTA into 10 µM Hsp70. Experiments were performed at 20 °C. The instruments stirring speed was set to 310 RPM and the feedback gain mode was set to “high”. Since the signal from the first injection can usually not be used for data analysis, only 2 µl were titrated in this step and the data point was omitted. Control titrations were performed by injecting the proteins into 35 mM HEPES buffer (pH 7.8) and the experimental data were corrected for dilution. Measured data were analyzed using the “Origin” software (MicroCal, Munich, Germany). Measurements were done in duplicate.

*RNA interference for Hsp70-depletion of HeLa cells and subsequent DT-intoxication*

RNA interference for Hsp70-depletion was performed as described^71^. For inhibition of endogenous Hsp70 protein expression, the siRNA corresponding to the Hsp70 mRNA sequence 5´-UGC ACC UUG GGC UUG UCU CCG UCG U-3´ was used (GE Dharmacon, Freiburg, Germany). The sequence 5´-UGC GUC GUC GAU CGC UUA CUC UCG U-3´ was used as control siRNA. Transfection of siRNA into HeLa cells was performed according to the manufacturer´s instructions. In brief, HeLa cells at about 50 % confluence were transfected with 200 nM siRNA using 0.5 µl lipofectamine 2000. After 4 h, the medium was exchanged and cells were further incubated for 48 h at 37 °C. Then, DT (69 nM) was added and DT-mediated cell-rounding as specific endpoint of the intoxication process was visualized. After 2 h, cells were lysed and sequential ADP-ribosylation of EF-2 was performed as described. Comparable input of proteins into the analysis and Hsp70-depletion were confirmed by immunostaining of actin or Hsp70, respectively.

*Microinjection*

Cells were microinjected with a Microinjector 5242, Micromanipulator 5171 and Femtotips (Eppendorf, Hamburg, Germany). p_c_ was 100 hPa, p_I_ was 160 hPa and t_I_ was 0.5 sec. We used an Alexa 568- or Alexa 488-conjugated secondary antibody as injection marker. Cells were left untreated for 30 min after microinjection. Subsequently, cells were treated with DT and subjected to time-lapse microscopy. For live-cell imaging, cells were incubated in a chamber with humidified atmosphere (6.5% CO_2_ and 9% O_2_) at 37 °C. Cells were analyzed with an Axio Observer microscope (Carl Zeiss, Jena, Germany), driven by Visiview imaging software (Visitron, Puchheim, Germany) with a 20x Fluar objectives, a CSU-X1 spinning disk confocal head (Yokogawa, Tokio, Japan) with emission filter wheel and a Coolsnap HQ II digital camera (PHOTOMETRICS, Tucson, AZ, USA) with 405-, 488-, and 561-nm laser lines. Time-lapse microscopy was stopped when ~40% of non-microinjected cells rounded up.

**Supplementary Figures**

**
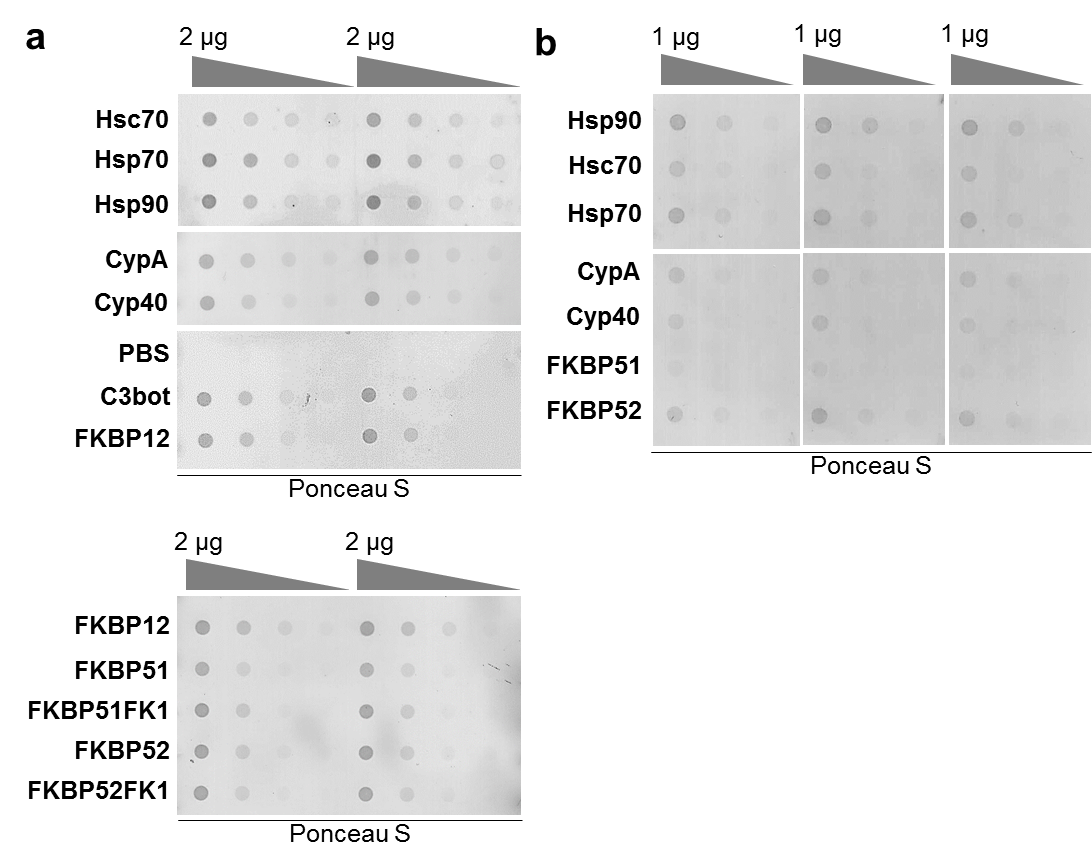
**

**Supplementary Fig. 1.** Ponceau S staining of the immobilized proteins analyzed in the Dot blot experiments shown in Fig. 1a (a) and Fig. 1b (b).

**
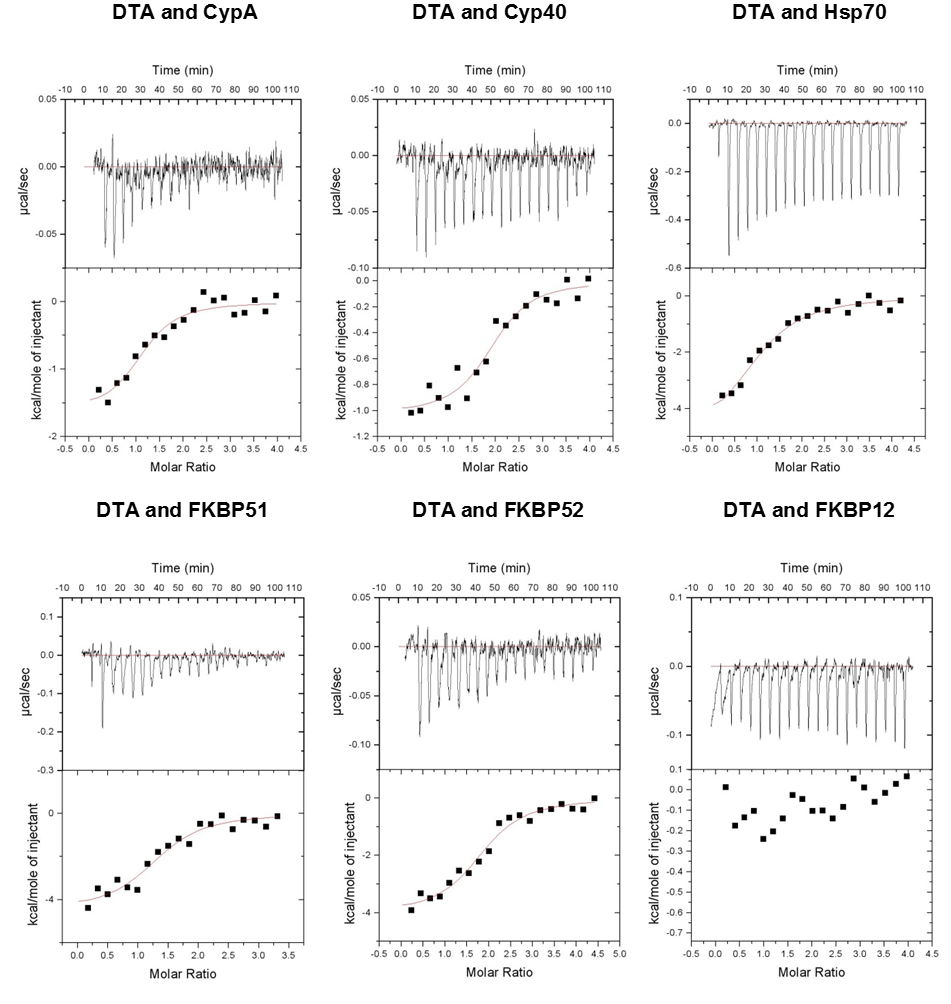
**

**Supplementary Fig. 2.** Isothermal titration calorimetry to determine the interaction between DTA and Hsp70 or the PPIases CypA, Cyp40, FKBP51, FKBP52 and FKBP12. For analyzing the interaction of DTA with PPIases by isothermal titration calorimetry, a solution of 10 μM DTA (initial concentration) in 35 mM HEPES buffer (pH 7.8) was titrated at 20 °C with 180 μM of the respective indicated PPIase. Analysis of the Hsp70/DTA interaction was performed by titration of a 190 µM solution of DTA into 10 µM Hsp70. Control titrations were performed by injecting the proteins into 35 mM HEPES buffer (pH 7.8) and the experimental data were corrected for dilution. Obtained data were fitted to a single-site binding model. The resulting thermodynamic parameters of each association reaction are listed in Supplementary Table 1.

**
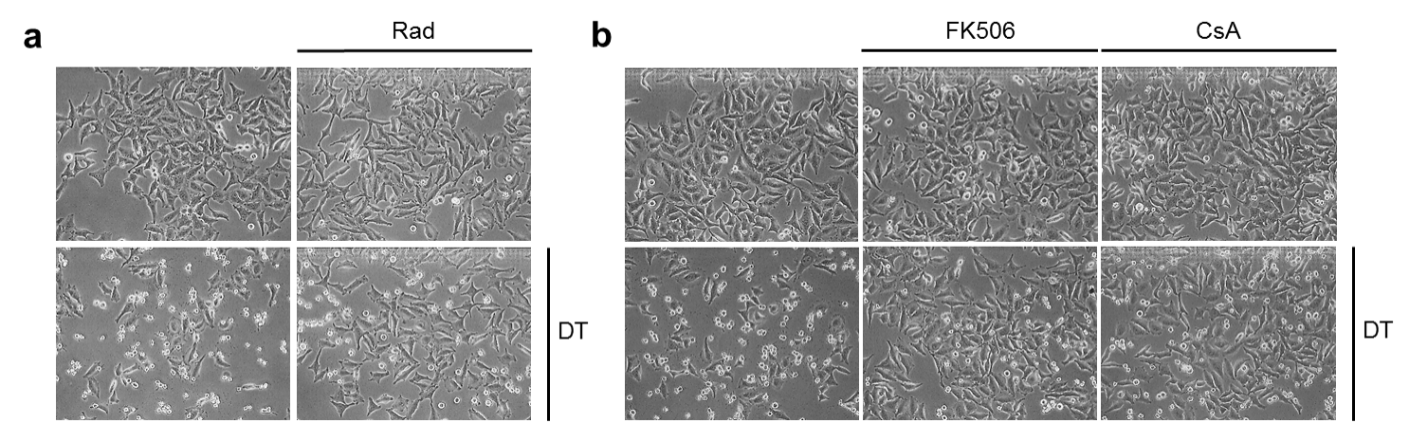
**

**Supplementary Fig. 3.** a. Representative pictures taken after 3.5 h of intoxication in the presence or absence of Rad. b. Representative pictures taken after 3 h of intoxication in the presence or absence of CsA and FK506. For quantitative analysis, the percentages of rounded cells were determined from the pictures.

**
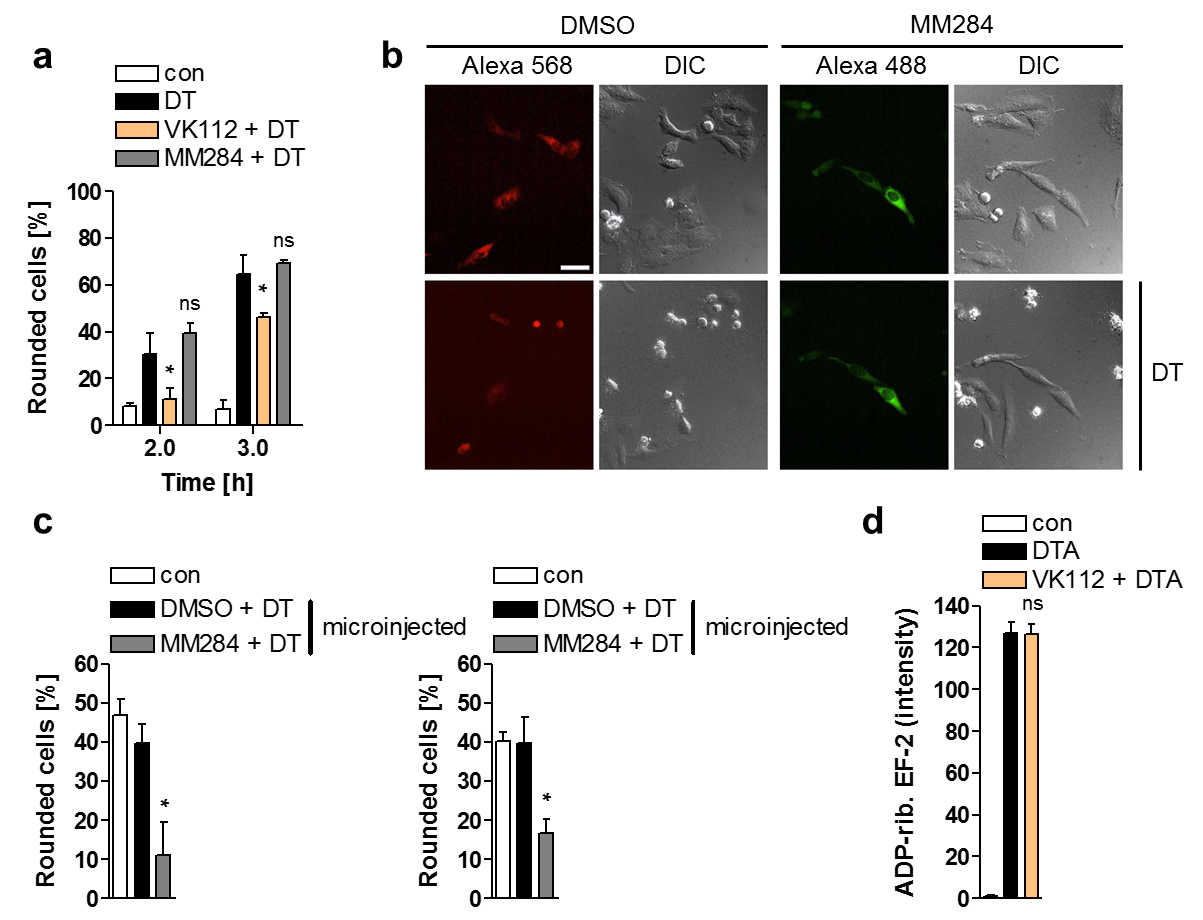
**

**Supplementary Fig. 4.** a. VK112 but not the non-cell-permeable derivative MM284 delays intoxication of HeLa cells with DT. Cells were pre-incubated for 30 min at 37 ˚C with either VK112 (200 µM) or MM284 (200 µM) or left untreated. DT (6.9 nM) was added and cells were further incubated. For control, cells were treated with DT alone or left untreated. After 2 and 3 h, pictures were taken to determine the percentage of round, i. e. intoxicated cells (mean ± SD; n = 3). Significance was tested between cells treated with DT in the absence (black bars) and presence of the respective inhibitor (colored bars) using Student´s t-test (ns, not significant, * p < 0.05).

b. HeLa cells were microinjected with MM284 (200 µM; green) or DMSO (red) as solvent control. 30 min after microinjection, cells were intoxicated with DT (69 nM) and subjected to time-lapse microscopy.

c. HeLa cells were microinjected as in b. After microinjection, cells were intoxicated with DT (8.6 nM (left panel) or 69 nM (right panel)) and subjected to timelapse microscopy. When ~40 % of non-microinjected cells rounded up the experiment was stopped. Left panel: Six fields of view (fov) were quantified for MM284 and five for DMSO (p < 0.05; mean ± SEM). Right panel: 24 fields of view (fov) were quantified for MM284 and 14 for DMSO (* p < 0.005; mean ± SEM).

d. Effect of VK112 on DTA-catalyzed ADP-ribosylation of EF-2 *in vitro*. Equal amounts of HeLa lysate protein (10 µg) were pre-treated for 30 min at 37 °C with VK112 (200 µM) or left untreated. Subsequently, DTA (100 ng) and biotin-labeled NAD^+^ (10 µM) were added and samples were incubated for 10 min at 37 °C. For control, a lysate sample was incubated without DTA. The reaction was stopped by boiling at 95 °C in SDS-sample buffer and proteins were separated by SDS-PAGE. Comparable amounts of the blotted proteins were confirmed by Ponceau S staining of the blot membrane. Biotinylated, i.e. ADP-ribosylated EF-2 was detected by Western blotting with streptavidin-peroxidase using the ECL system. For comparison of samples, the intensity of biotin-labeled EF-2 was evaluated via densitometry.

**
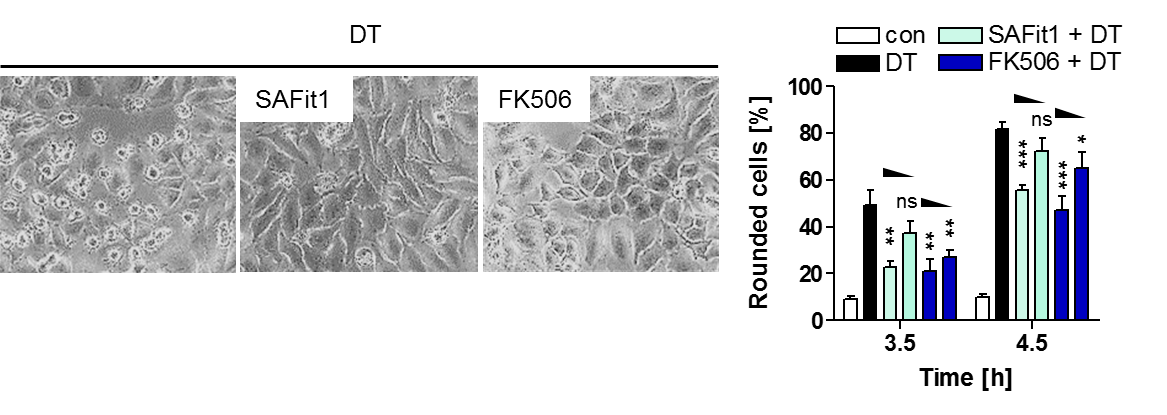
**

**Supplementary Fig. 5.** Effect of the FKBP51-selective inhibitor SAFit1 on the intoxication of HeLa cells with DT. HeLa cells were pre-treated for 30 min with either the non-selective FKBP inhibitor FK506 or the FKBP51-selective inhibitor SAFit1 (15 and 30 µM) and challenged with DT (0.43 nM) and the intoxication of the cells after 3.5 and 4.5 h was monitored in terms of cell-rounding. The pictures shown in the left panel were taken after 3.5 h and the percentage of round, i. e. intoxicated cells was determined (mean ± SD; n = 3). Significance was tested between cells treated with DT in the absence (black bars) and presence of the respective inhibitor (colored bars) using Student´s t-test (ns, not significant, * p < 0.05, ** p < 0.01, *** p < 0.001).

**
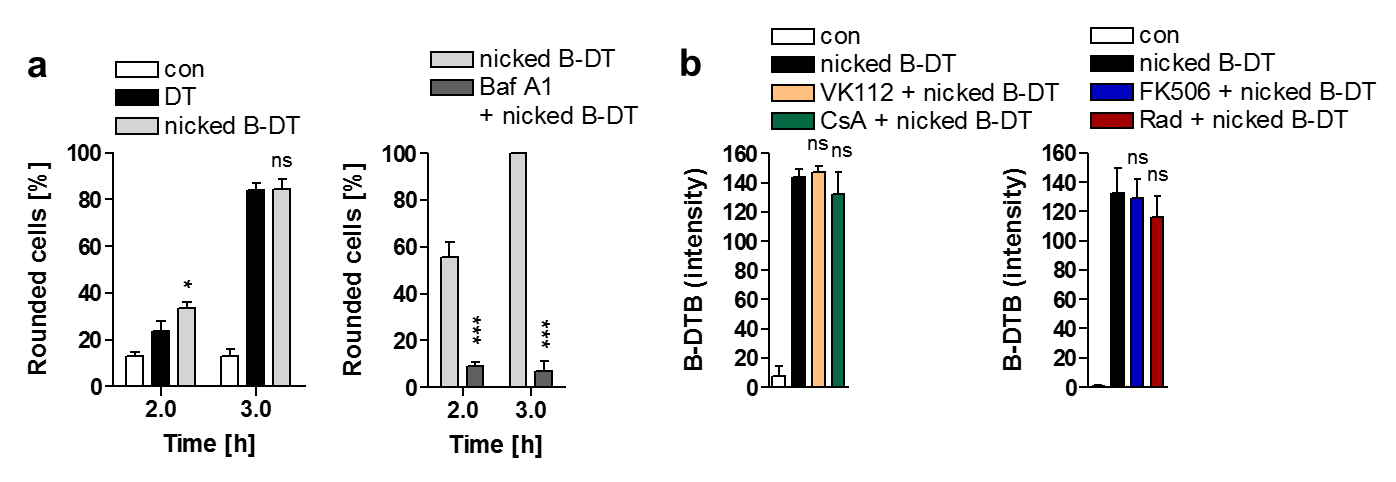
**

**Supplementary Fig. 6.** a. Nicked biotinylated DT (nicked B-DT) is biologically active. HeLa cells were incubated at 37 ˚C with equal final concentrations (8.6 nM) of DT or nicked B-DT. After 2 and 3 h, pictures were taken to determine the percentage of round, i. e. intoxicated cells (mean ± SD; n = 3). Significance was tested between cells treated with DT (black bars) and nicked B-DT (grey bars) or nicked B-DT in the absence (light grey bars) and presence of Baf A1 (dark grey bars) using Student´s t-test (ns, not significant, * p < 0.05, *** p < 0.001). Less cells rounded up when they were pre-treated with 100 nM Baf A1 prior to toxin application.

b. CsA (and its derivative VK112), FK506 or Rad do not inhibit binding of nicked DT to the surface of HeLa cells. Cells were pre-incubated for 30 min at 37 °C with either Rad (20 µM), CsA (20 µM), the non-immunosuppressive CsA-derivative VK112 (200 µM) or FK506 (10 µM), or left untreated for control. Then, cells were incubated for 15 min at 4 °C in serum-free medium with biotin-labeled nicked DT (6.9 nM) to enable its binding to the cell receptors. After washing, cells were lysed in sample buffer and subjected to SDS-PAGE. The cell-associated biotinylated DTB was detected by Western blotting with streptavidin-peroxidase. The intensity of biotin-DTB was evaluated via densitometry to estimate the amount of bound toxin. Values are given as the mean ± SD (n = 3); significance was tested between cells treated with nicked B-DT in the absence (black bars) and presence of the respective inhibitor (colored bars) using Student´s t-test (ns, not significant).

**
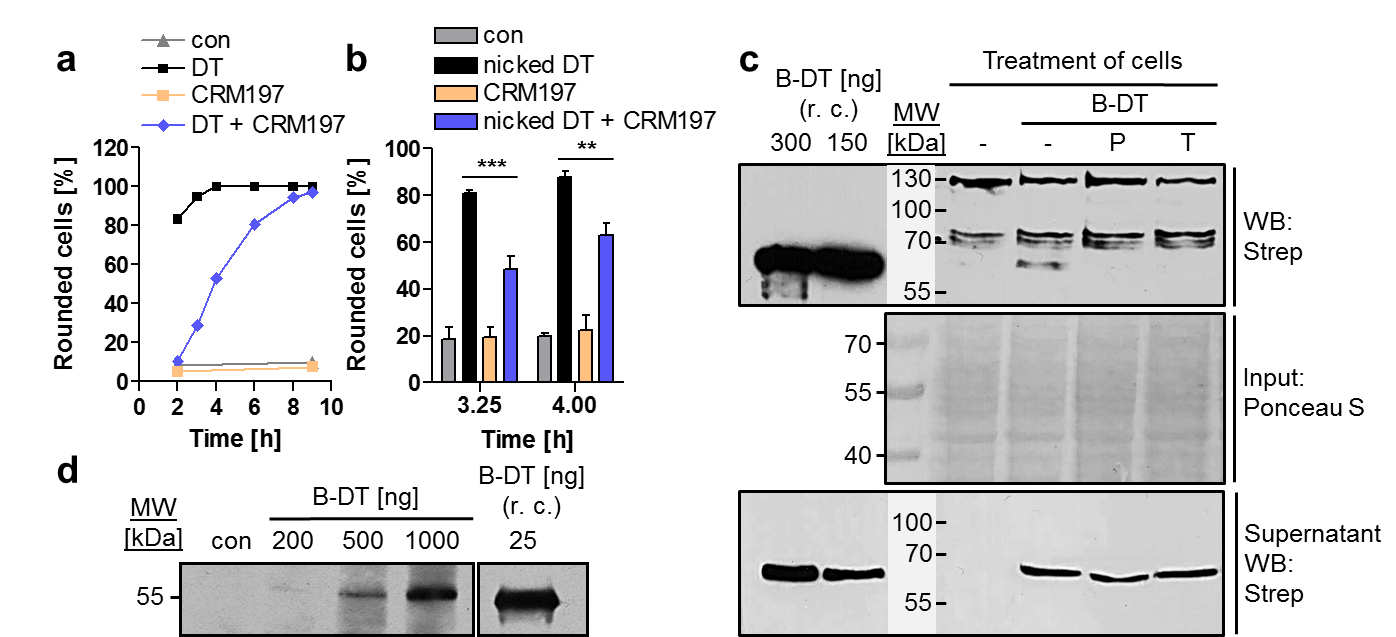
**

**Supplementary Fig. 7.** a. DT competes with CRM197 for cellular uptake. HeLa cells were incubated at 37 ˚C with DT (17.25 nM), CRM197 (172.5 nM) or a combination of both toxins. Cellular uptake of DT was monitored by microscopy and quantified by calculating the percentage of round cells (mean ± SD; n = 3).

b. DT competes with CRM197 for cell surface binding. HeLa cells were incubated with nicked DT (17.25 nM), CRM197 (172.5 nM) or a combination of both toxins for 30 min at 4 °C. Then, medium was exchanged and cells were further kept at 37 °C. Pictures were taken after 3.25 and 4 h to calculate the percentage of round cells (mean ± SD; n = 3). Significance was tested between cells treated with nicked DT (black bars) and the combination of both toxins (blue bars) using Student´s t-test (** p < 0.01, *** p < 0.001).

c. Proteolytic digestion of cell surface proteins prevents binding of biotin-labeled DT. HeLa cells were pre-treated for 30 min at 37 °C with pronase (P; 500 µg/ml) or trypsin (T; 100 µg/ml) or left untreated for control. Subsequently, serum or trypsin inhibitor was added to cells to block the proteolytic activity prior to washing of the cells and incubation for 10 min at 4 °C with biotin-labeled DT (34.5 nM). Control cells were left untreated. Cells were lysed after washing and submitted to Western blot analysis against biotinylated proteins. To exclude proteolytic digestion of biotin-labeled DT in the supernatant of pronase- or trypsin-treated cells, the supernatant was collected after incubation on ice and immunoblotting against biotin-labeled proteins was performed using streptavidin-peroxidase.

d. Determination of the amount of biotinylated DT for sufficient detection of bound B-DTB on the cell surface. Cells were kept at 4 °C for 15 min followed by incubation for 30 min at 4 °C with different amounts of biotin-labeled DT (200 ng, 500 ng and 1,000 ng) to enable DT-binding to the cell receptors. For control, cells were left untreated. After washing, cells were lysed in sample buffer and subjected to SDS-PAGE. The biotinylated DT was detected by Western blotting with streptavidin-peroxidase. B-DT (25 ng) was used as running control (right lane).

**
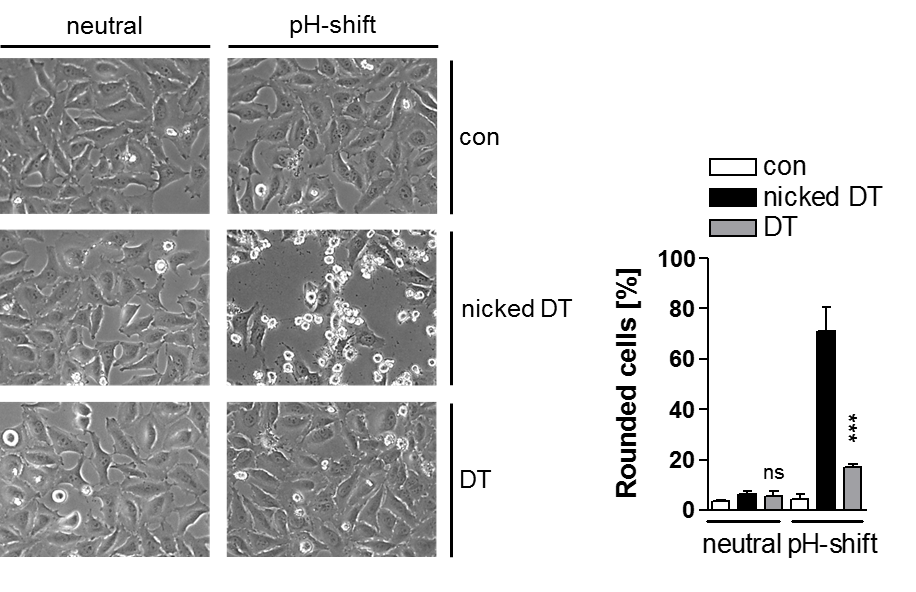
**

**Supplementary Fig. 8.** HeLa cells were pre-incubated for 30 min at 37 °C with 100 nM Baf A1 to block the normal uptake of DTA into the cytosol and then incubated with either nicked DT (13.8 nM) or non-activated DT (13.8 nM) for 10min at 4 °C in serum-free medium to allow toxin-binding to the cell surface. Then, the medium was adjusted to pH 4.5 and the cells were incubated at 37 °C for further 15 min (pH-shift). For control, cells were incubated for 15 min with neutral medium instead of the pH-shift. Subsequently, the cells were further incubated in warm and neutral serum-containing medium containing BafA1 and after 4 h pictures were taken (left panel) and the percentage of rounded cells was determined from the pictures (right panel). Values are given as mean ± SD (n = 3). Significance was tested between cells treated with nicked DT (black bars) and DT (grey bars) using Student´s t-test (ns, not significant, *** p < 0.001).

**
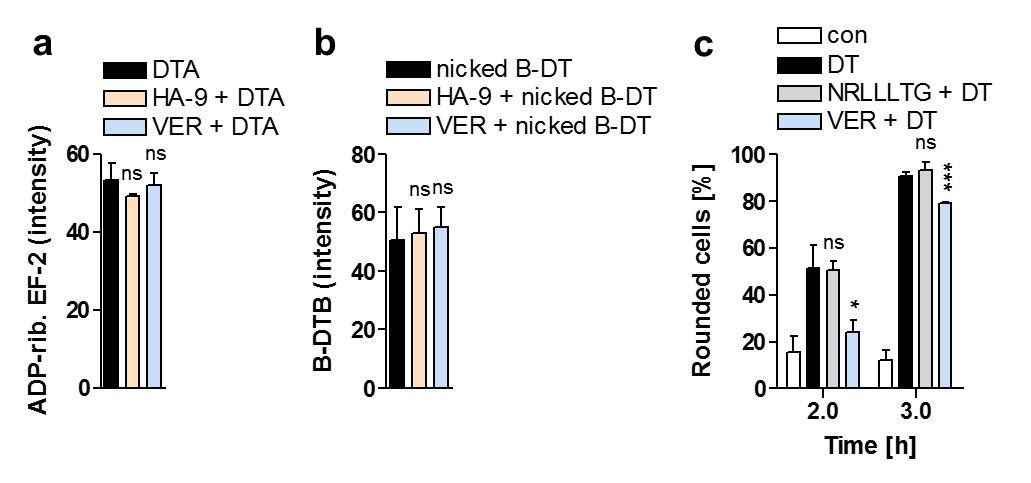
**

**Supplementary Fig. 9.** a. Effect of VER155008 and HA-9 on the DTA-catalyzed ADP-ribosylation of EF-2 *in vitro*. Equal amounts of HeLa lysate protein (10 µg) were pre-treated for 30 min at 37 °C with or without VER155008 (VER, 20 µM) or HA-9 (20 µM) and then DTA (100 ng) and biotin-labeled NAD^+^ (10 µM) were added. A lysate sample was incubated without DTA for control (not shown). After 10 min incubation at 37 °C, the reaction was stopped by boiling the samples at 95 °C in SDS-sample buffer and the proteins were separated by SDS-PAGE. Comparable amounts of the blotted proteins were confirmed by Ponceau S staining of the blot membrane (not shown). The biotinylated, i.e. ADP-ribosylated EF-2 was detected by Western blotting with streptavidin-peroxidase using the ECL system and the intensity of biotin-labeled EF-2 evaluated via densitometry.

b. HA-9 and VER155008 do not inhibit binding of nicked DT to HeLa cells. Cells were pre-incubated for 30 min at 37 °C with either HA-9 (20 µM) or VER155008 (VER, 20 µM) or left untreated. Subsequently, cells were incubated for 15 min at 4 °C in serum-free medium with biotin-labeled nicked DT (13.8 nM) to enable its binding to the cell receptors. For control, cells were incubated without toxin (not shown). After washing, cells were lysed in sample buffer and subjected to SDS-PAGE. The cell-associated, biotinylated DTB was detected by Western blotting with streptavidin-peroxidase. The intensity of biotin-DTB was evaluated via densitometry to estimate the amount of bound toxin.

c. Effects of the non-cell-permeable Hsp70-inhibiting peptide NRLLLTG (20 µM) and the cell-permeable Hsp70/Hsc70-inhibitor VER155008 (20 µM) on the intoxication of HeLa cells treated for 2 h with DT (0.86 nM). Values are given as the mean ± SD (n = 3); significance was tested between cells treated with DT in the absence (black bars) and presence of the respective inhibitor (colored bars) using Student´s t-test (ns, not significant, * p < 0.05, *** p < 0.001).

**
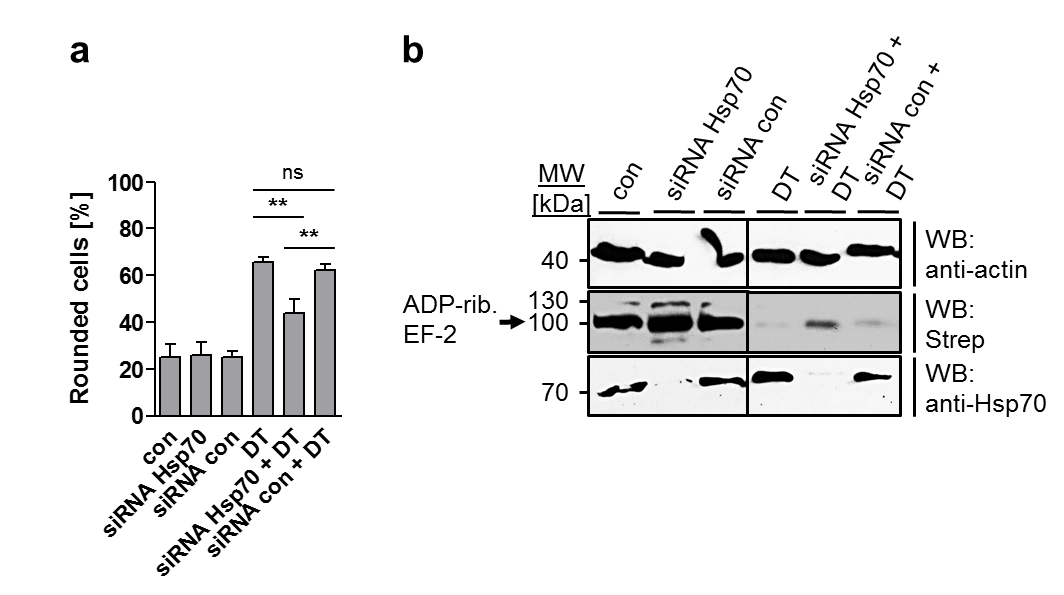
**

**Supplementary Fig. 10.** Effect of Hsp70-depletion on intoxication of cells with DT. HeLa cells were transfected with siRNA Hsp70 or siRNA control (siRNA con) or left untreated (con). 48 h after transfection, DT (69 nM) was added. For control, cells were incubated without DT (con, siRNA Hsp70 and siRNA con). a. After 2 h, pictures were taken to determine the percentage of rounded cells. Values are mean ± SD (n = 3); significance was tested using Student´s t-test (ns, not significant, ** p < 0.01). b. Thereafter, cells were lysed and equal protein amounts incubated for 30 min at 37 °C with biotin-NAD^+^ (10 µM) and DTA (100 ng). Biotinylated (i.e. ADP-ribosylated) proteins were detected by Western blotting with streptavidin-peroxidase to analyze the ADP-ribosylation of EF-2. Comparable input of proteins into the analysis was confirmed by determination of the actin content (upper panel). Hsp70-depletion of the respective sample was shown via immunostaining of Hsp70.

**Supplementary Tables**

**Supplementary Table 1. The thermodynamic parameters characterizing the binding of DTA to the PPIases CypA, Cyp40, FKBP51 and FKBP52, as determined by isothermal titration calorimetry (ITC).** For analyzing the interaction of DTA with PPIases by isothermal titration calorimetry, a solution of 10 μM DTA (initial concentration) in 35 mM HEPES buffer (pH 7.8) was titrated at 20 °C with 100 μM of the respective indicated PPIase. Analysis of the Hsp70/DTA interaction was performed by titration of a 190 µM solution of DTA into 10 µM Hsp70. Results were fitted to a single-site binding model, the reported errors correspond to the s.d. of the fit. The original results are shown in Supplementary Fig. 2. (n.b., no binding detectable). Analysis was performed in duplicate with similar results. Stoichiometries for FKBP51, CypA and Hsp70 suggest 1:1 binding to DTA. For Cyp40 and FKBP52 data suggest 2:1 binding to DTA, however incorrect folding into a non-functional state of the folding helpers can not be ruled out.

| **Folding helper** | **K_D_**  **(µM)** | **ΔH**  **(kcal/mol)** | **-TΔS**  **(kcal/mol)** | **ΔG**  **(kcal/mol)** | **n** |
| --- | --- | --- | --- | --- | --- |
| CypA | 1.4 ± 0.65 | -1.6 ± 0.2 | -6.2 | -7.8 | 1.1 |
| Cyp40 | 0.94 ± 0.21 | -1.1± 0.06 | -7.0 | -8.1 | 1.9 |
| FKBP51 | 1.3 ± 0.2 | -4.5 ± 0.4 | -3.4 | -7.9 | 1.3 |
| FKBP52 | 0.74 ± 0.21 | -4.0 ± 0.2 | -4.2 | -8.2 | 1.8 |
| Hsp70 | 3.5 ± 0.9 | -5.2 ± 0.6 | -2.1 | -7.3 | 1.1 |
| FKBP12 | n.b. | n.b. | n.b. | n.b. | n.b. |
